# Causal Mediation and Functional Outcome Analysis with Process Data

**DOI:** 10.1017/psy.2026.10087

**Published:** 2026-01-23

**Authors:** Youmi Suk, Chan Park

**Affiliations:** 1 Teachers College, Columbia University, USA; 2 University of Illinois Urbana-Champaign, USA

**Keywords:** causal inference, causal mediation, functional data, generalized additive models, process data

## Abstract

Over the past two decades, there has been growing interest in analyzing the effects of educational programs on outcomes using process data from computer-based testing and learning environments. However, most analyses focus on final outcomes at the end of a test or session, overlooking their functional nature over time and neglecting causal mechanisms. To address this gap, this article proposes a novel causal mediation framework for identifying and estimating functional natural direct effects, functional natural indirect effects, and functional total effects, along with their subgroup effects. We define these effects using potential outcomes and provide nonparametric identification strategies depending on whether post-treatment covariates are present or not. We then develop estimation methods using generalized additive models, a flexible and robust tool for analyzing functional data. Through a simulation study, we assess the finite-sample performance of the proposed approach by comparing it to parametric regression methods. We also demonstrate our approach by examining the effects of extended time accommodations on two functional outcomes using process data from the National Assessment of Educational Progress. Our mediation approach with functional outcomes effectively captures dynamic causal mechanisms underlying the program’s effects and pinpoints when and for whom each effect manifests throughout the testing period.

## 1 Introduction

The transition to computer-based testing and digital assessments in various testing programs began in the early 2000s. Recent technological advancements and evolving educational needs have driven the widespread use of these digital assessments (Bennett et al., 2007; Thurlow et al., 2010). A key feature of digital assessments is the collection of *process data*—information on all of the responses about test-takers’ interactions with computer devices during the test. Process data include real-time responses and actions, such as mouse clicks, keystrokes, response times, and behaviors related to testing accommodations (Bergner & von Davier, 2018). Not surprisingly, the rich and detailed information from these data helps researchers understand test-takers’ problem-solving behaviors and their engagement with accessibility tools and testing accommodations. While research using process data has been actively conducted for tasks, such as ability estimation and cheating detection (Jiao et al., 2021), its methods and applications to causal inquiry are still in their early stages. The overarching goal of this article is to propose a causal framework with time-stamped process data to assess the dynamic causal effects of test-related or educational programs.

As a concrete example, consider evaluating the effect of extended time accommodations (ETAs), the most frequently provided accommodation to students with disabilities and/or English language learners. Previous studies (Lee & Suk, 2025; Suk & Kim, 2025; Wei & Zhang, 2023) used process data to identify whether students who received ETA made use of it during a test and, with this variable, evaluated the effects of using ETA on test scores through different quasi-experimental designs (see Section 2.1 for details). However, these studies focus on test-takers’ final outcomes measured at the end of a test or session and overlook their functional nature over time. It is possible that ETA treatment groups may not differ in terms of final outcomes, but may show temporal or consistent differences during specific periods of testing. Capturing such changes requires researchers to analyze evolving test scores over time rather than the final test scores and view the former as *functional outcomes* from the field of functional data analysis (Ramsay & Silverman, 2005; Wang et al., 2016). Briefly, functional data are defined as data that change over a continuous domain (e.g., time and space), and each unit’s outcome is typically a function or a curve that varies over a continuum rather than a single discrete point. Regression models for such functional outcomes try to capture evolving patterns like time-dependent processes or two-dimensional images (Ramsay & Silverman, 2005) (see Section 2.2 for more details on functional data). However, there is still an open question as to how to analyze process data in a functional format to draw causal inferences.

Moreover, the rich information from process data has the potential to uncover the *causal mechanisms* in digital assessments, i.e., how and why educational programs influence an outcome. For example, previous studies on the ETA evaluation (e.g., Lee & Suk, 2025; Suk & Kim, 2025) assume that the effect of ETA on an outcome manifests only by using it. However, changes in outcomes may result either from using ETA (i.e., indirect effect) or from receiving ETA itself, not through its actual use (i.e., direct effect). This direct effect captures some psychological benefits of receiving ETA, even if the ETA itself is not actually used. For example, the ETA availability can positively affect student performance by reducing anxiety (Lovett & Leja, 2013). The direct and indirect effects may also vary depending on subgroups defined by test-takers’ characteristics (e.g., disability status and gender). Investigating both direct and indirect effects and conducting their subgroup/moderation analysis helps gain a more nuanced understanding of program effects. While existing causal tools on causal mediation (e.g., Pearl, 2001) and subgroup analysis (e.g., Yang et al., 2021) can support such investigations, a systematic methodology is still needed to extend these tools to the functional data context, in order to capture dynamic aspects of direct and indirect effects using process data in a functional format.

This article proposes a novel causal mediation framework with a functional outcome to evaluate test-related or educational programs in process data. The proposed framework provides the counterfactual definitions for the functional natural direct and indirect effects, and their total average effect. For subgroup analysis, we define these functional effects among subgroups determined by a subset of pre-treatment covariates. We also provide nonparametric identification strategies for each effect depending on the presence or absence of post-treatment confounders and construct estimation methods using generalized additive models (GAMs; Hastie & Tibshirani, 1986), a flexible and robust tool for analyzing functional data. Through a simulation study, we demonstrate the effectiveness of the proposed GAM-based methods by comparing them with parametric regression methods. Furthermore, we apply our framework to examine the effects of ETA on two functional outcomes—evolving test scores and evolving item access—using process data from the National Assessment of Educational Progress (NAEP).

The remainder of the article is organized as follows. Section 2 reviews the relevant literature and highlights our contributions. Section 3 describes the study setup. Section 4 outlines our causal mediation framework with a functional outcome by building on the existing causal mediation literature. Section 5 describes the design and results of our simulation study, and Section 6 demonstrates our framework to evaluate the effects of ETA in the NAEP process data. Finally, Section 7 discusses the implications and conclusions of this study.

## 2 Prior work and our goals

### 2.1 Causal inference with process data

Most of the research efforts using process data from digital assessments have focused on estimating ability parameters, facilitating cognitive diagnosis, and detecting aberrant responding behavior (Jiao et al., 2021). However, only a few studies have used process data to explore causal questions about students’ testing behaviors or test-related programs. For example, Wei and Zhang (2023) use a propensity score method to balance covariates across three distinct profiles of students with learning disabilities: those who do not receive ETA, those who receive ETA but do not use it, and those who both receive and use ETA. They construct students’ ETA use status based on the NAEP process data by comparing their total response time with the standard testing time, and then analyze the math performance among the three profiles. However, Suk and Kim (2025) suggest using a fuzzy regression discontinuity design with multiple control groups to evaluate the effects of using ETA near the cutoff of a running variable. In their approach, the ETA eligibility status serves as an instrumental variable, and the effect bounds are constructed using multiple control groups. More specifically, the running variable is the English proficiency levels of English language learners, and multiple control groups occur because students either do not receive ETA or decline to use it when offered. Using the NAEP process data, they construct the ETA use status similarly to Wei and Zhang (2023) and find no strong evidence of using ETA on the final test scores among a subpopulation.

Additionally, Lee and Suk (2025) propose an evidence factor analysis in fuzzy regression discontinuity designs to reinforce causal conclusions on the effect of using ETA within a single dataset. Evidence factors represent two or more independent tests of the same null hypothesis about the treatment effect within a single dataset, each potentially subject to different biases (Rosenbaum, 2010). They also use the NAEP process data to demonstrate how to construct and combine three evidence factors in the ETA example, and as a result, there is no strong evidence of the effect of using ETA on the final test scores.

Although these prior studies have utilized process data to find causal evidence on students’ behaviors or testing programs, they have only incorporated a limited portion of process data into causal research, without fully using time information. These studies also assume that the treatment effect manifests only by using it and ignore the potential direct effect of receiving it, not mediated by its actual use. Therefore, there is a need to develop a rigorous methodology for analyzing time-stamped process data to draw causal inferences and discover the underlying mechanisms.

### 2.2 Causal inference with functional data

In classical statistical methods, the fundamental observational unit is typically a scalar or a vector of fixed length. However, with advances in technologies, observational units can take more complex forms, such as functions, curves, images, or other objects that vary over a continuum. Functional data analysis is a branch of statistics specifically developed to handle such non-scalar data. For simplicity, we will refer to these complex objects as “functions” throughout the manuscript. For example, in NAEP process data, one might traditionally analyze only the final math test score using classical statistical methods, say using a linear regression that regresses the final test score on other covariates. However, if process data capture the *entire trajectory* of the evolving test score over time, it can be viewed as a function over the testing period. Such functional observations can preserve the full temporal dynamics of test-taking processes.

With the shift in the observational unit, the goals of functional data analysis include understanding relationships, making predictions, and identifying patterns with functional variables. In this field, methodological advances have been made to more effectively address the high dimensionality and inherent complexity of functional data. Key developments include functional principal component analysis, functional regression with functional outcomes and/or functional predictors, and clustering of functional data (Crainiceanu et al., 2024; Ramsay & Silverman, 2005; Wang et al., 2016). However, most existing studies are limited to exploring associations rather than causal relationships, and the development of causal inference techniques with such functional data is in its infancy.

Recently, some notable work has been developed. Ecker et al. (2023) introduce a causal framework with a functional outcome (e.g., cumulative lifetime incomes) and a scalar binary treatment (e.g., residing in urban versus rural areas) to target the functional average treatment effect (FATE), i.e., the average treatment effect (ATE) over the functional domain. They extend the standard causal assumptions (consistency, positivity, and ignorability/unconfoundedness) to the functional data context to identify the FATE. Ecker et al. (2023) use functional linear regression as the outcome model to estimate the FATE and develop simultaneous confidence bands for valid inference across the functional domain. They also examine the subgroup effects of FATE to find effect heterogeneity, say between males versus females. In contrast, Gao et al. (2024) propose a causal framework with a functional treatment (e.g., hippocampal changes over time) and time-to-event (survival) outcomes (e.g., time until conversion from mild cognitive impairment to Alzheimer’s disease). They define the target estimand as the coefficient of functional treatment in a type of functional linear regression model and provide three estimation methods, including regression methods, weighting methods, and combinations of these.

Since process data are collected over a specific time period, it can be considered a type of functional data. As such, causal inference methods for functional data could be applied to evaluate the effects of students’ testing behaviors or test-related programs on the functional outcomes, such as evolving test scores. However, we need additional considerations to accurately establish a functional causal framework for process data. For example, process data from digital-based assessments may require additional assumptions for causal identification due to the unique nature of assignment and outcome processes in digital environments (e.g., one-sided noncompliance and constant potential outcomes over a certain period). Furthermore, while existing studies (Ecker et al., 2023; Gao et al., 2024) focus on estimating the FATE (or a type of ATE), they are limited in investigating causal mechanisms through mediators across the functional domain. In cases where researchers wish to decompose the total effect into direct and indirect effects in functional data, they need to develop a new framework and methods for causal mediation with functional data.

### 2.3 The goals of this article

To address the limitations of existing approaches, this article seeks to develop a new causal mediation framework for functional outcomes derived from process data. Specifically, we pursue the following four goals.

First, we aim to re-conceptualize process data as functional outcomes. While researchers in education and psychology often interpret evolving outcomes (e.g., test scores recorded during a test) as repeated discrete outcomes within a longitudinal framework, we adopt a functional data perspective. Under this paradigm, evolving test scores are treated as realizations of an underlying continuous process, where the study unit is an individual’s entire outcome trajectory rather than time-point measurements. This functional view is more appropriate for process data and marks a conceptual shift that is relatively new to the fields of psychology and education. We illustrate this shift using a motivating example of ETA, which serves as the empirical context throughout the manuscript.

Second, we aim to define causal estimands that uncover dynamic causal mechanisms over a functional domain (e.g., time or space). Specifically, we define the average functional indirect and direct effects, along with their subgroups effects, where the subgroups are defined by a subset of pre-treatment covariates (see Equations (1)–(4) for details). The resulting framework enables researchers to effectively answer the causal question: “*Why, when, and for whom does a program work?*” The “*why*” component concerns causal mechanisms through direct and indirect pathways; the “*when*” component captures how effects evolve over the functional domain; and the “*whom*” component addresses subgroup effects beyond the ATE. When the functional domain corresponds to space, our framework can also address *where* a program is effective.

Third, we aim to establish identification strategies under several realistic scenarios, including: (i) the presence or absence of post-treatment confounders, (ii) mediators derived from other variables, and (iii) one-sided versus two-sided noncompliance. In particular, the presence of post-treatment confounders requires jointly modeling the mediator and post-treatment confounders. We outline the identification assumptions that are most relevant to our process data setting in the main text, while providing detailed identification results for each scenario in the appendices of the Supplementary Material to broaden the applicability of our approach.

Fourth, we aim to develop estimation and inference procedures for functional causal effects using GAMs. GAMs are widely used in functional data analysis due to their flexibility and adaptivity (Crainiceanu et al., 2024; Ramsay & Silverman, 2005). We leverage GAMs to construct estimators for our functional direct and indirect effects. Importantly, our estimators go beyond fitting outcome regression by incorporating carefully designed weighting schemes to account for post-treatment variables. For inference, we employ a cluster bootstrap that resamples individuals, rather than time points, to consider unit-specific serial dependence. Through simulation studies, we demonstrate that our GAM-based methods achieve a favorable bias–variance trade-off and perform at least as well as correctly specified parametric methods.

## 3 Setting

### 3.1 Notation

Let i=1,⋯,n index subjects, i.e., study units. We suppress the subscript *i* unless necessary. We consider a setting where the following variables are available for subject *i* from functional data. We denote the treatment receipt variable as Z∈{0,1} , where Z=1 indicates that a subject received the treatment and Z=0 indicates that the subject did not receive it. We denote a set of pre-treatment covariates as $X=\{X_{1},\cdots ,X_{p}\}\in R^{p}$ . We also denote the observed outcome process as $Y(K)=\{Y(k):k\in K=k_{0},k_{m}\}$ , where $k_{0}$ and $k_{m}$ represent the minimum and maximum values of the functional domain K , respectively. The minimum value $k_{0}$ plays a key role in the definition and identification of the functional causal effects (see Section 4 for details). Additionally, $W=\{W_{1},\cdots ,W_{q}\}\in R^{q}$ denotes the observed post-treatment covariates by time $k_{0}$ (e.g., the number of accessed items up to $k_{0}$ ).

For causal mediation analysis with functional data from digital assessments, we introduce additional variables. Let T∈{0,1} denote the actual treatment use, where T=1 indicates that a subject used the treatment and T=0 indicates that the subject did not use it. Here, *T* and *Z* differ because subjects who receive the treatment can decide whether to use it, and *T* serves as a mediator. We denote a subject’s total response time as H∈K . In particular, we observe the outcome process Y(k) over $k\in k_{0},H$ , where the last observation ends at time *H*, and we use *H* to derive the treatment use variable as 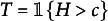. Therefore, the observed data for a subject consists of $\left(X,Z,T,H,W,Y(k_{0},H)\right)$ (see Table 1 for the structure of the process data). In Table 1, the first subject ( i=1 ) has three covariates $\left\{X_{1},X_{2},X_{3}\right\}$ with values {1, 0, 0}, an ETA receipt status of Z=0 (did not receive), an ETA use status of T=0 (did not use), and a total response time of H=5 minutes. Under $k_{0}=3$ , this student’s post-treatment covariate is observed as W=2 . Their functional outcome *Y* is observed from $k_{0}=3$ to their total response time *H*, with values of 1, 2, and 3 at k=3,4 , and 5 minutes, respectively.

**Table 1:** An example of the process data with three-dimensional $X=\{X_{1},X_{2},X_{3}\}$ , one-dimensional *W*, and $k_{0}=3$ Table 1 long description.

*i*	$X=\{X_{1},X_{2},X_{3}\}$	*Z*	*T*	*H*	*W*	Y({3,4,⋯,H})
1	{1,0,0}	0	0	5	2	{1,2,3}
2	{0,1,1}	1	0	4	1	{1,2}
⋮	⋮	⋮	⋮	⋮	⋮	⋮
*n*	{1,1,1}	1	1	7	1	{0,1,2,2,3}

**Figure 1:**
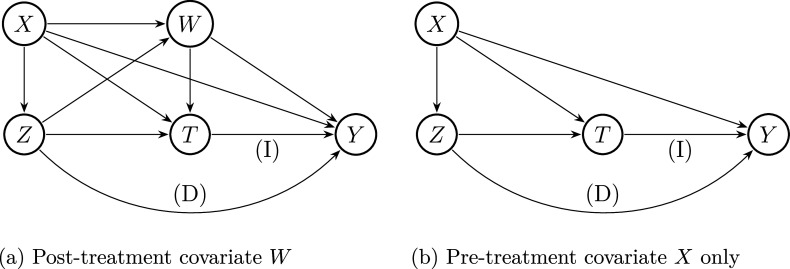
Graphical representations of causal relationships with a functional outcome. The arrows labeled with (D) and (I) represent the functional direct effect and functional indirect effect, respectively. Figure 1 long description.

Following the Neyman–Rubin’s potential outcome framework (Neyman, 1923; Rubin, 1974) and its extension to causal mediation (Pearl, 2001; Robins & Greenland, 1992), we define potential (also known as counterfactual) variables. Let $T^{z}(k)$ denote the potential treatment use (i.e., mediator) if *Z* were set to Z=z at time *k*. The relationship between treatment *Z* and mediator *T* can be either two-sided or one-sided. For two-sided noncompliance, we assume that $T^{z}(k)\in \{0,1\},z=0,1$ , but for one-sided noncompliance, we assume that $T^{1}(k)\in \{0,1\}$ and $T^{0}(k)=0$ . Similarly, let $W^{z}$ be a subject’s potential post-treatment covariate if they received the treatment Z=z . This implicitly assumes that the post-treatment covariate *W* is not affected by the treatment use *T*, but *W* influences *T* (see Figure 1 in the next section for a graphical illustration). Lastly, let $Y^{z,T^{z'}}(K)=\{Y^{z,T^{z'}(k)}(k):k\in K\}$ denote a subject’s potential outcome process if *Z* were set to *z* and *T* were set to the value it would naturally take under Z=z' . For example, $Y^{1,T^{0}}(K)$ indicates the potential outcome process if a subject received the treatment (i.e., Z=1 ) and their mediator *T* took the value it would have attained under the control condition (i.e., Z=0 ).

### 3.2 Motivating example

Testing accommodations are essential for students with disabilities and English language learners to fully demonstrate their abilities during a test. The ETA is the most frequently provided testing accommodation in many testing programs. Suppose that a researcher is interested in studying the causal mechanism and determining whether the benefit of ETA is a consequence of using ETA (i.e., indirect effect) or partially attributed to receiving ETA, not mediated via its actual use (i.e., direct effect). We also observe one-sided noncompliance in our ETA example; only students who receive ETA can decide to use it or not, but those who do not receive ETA cannot use it.

Figure 1 visualizes the assumed causal mechanisms of ETA with the receipt status (*Z*) and use status (*T*). Figure 1a describes the case where post-treatment covariates *W* are present, and Figure 1b describes the case only with pre-treatment covariates *X*. Confounding between *T* and *Y* arises due to students’ decisions about whether to use ETA, influenced by *Z*, *X*, and, if applicable, *W*. The total effect of *Z* on functional outcome *Y* can be decomposed into the indirect causal pathway mediated by *T* (i.e., Z→T→Y ) and the direct pathway not mediated by *T* (i.e., Z→Y ).

## 4 Causal mediation with a functional outcome from process data

In this section, we extend the standard causal mediation framework (e.g., Pearl, 2001) to the functional outcome context, particularly using process data in digital-based assessments. We use the ETA in digital-based assessments as an illustration of the proposed framework. In this setting, we observe one-sided noncompliance between treatment *Z* and mediator *T* (i.e., $T^{1}(k)\in \{0,1\}$ and $T^{0}(k)=0$ ). Additionally, to clearly interpret the causal estimands below, we make the following assumption.

Assumption A1 **(Stabilized decision).**

- There exists a constant $k_{0}$ such that $T^{z}(k)$ is constant for $k_{0}\leq k$ , i.e., $T^{z}(k)=T^{z}(k')$ if $k_{0}\leq k,k'$ . Therefore, we simply denote $T^{z}=T^{z}(k)$ for $k_{0}\leq k$ .

Assumption A1 states that a student’s decision to use the offered ETA becomes constant at or after the minimum valid time $k_{0}$ . For example, if the minimum valid time is 10 minutes after the test starts, it is assumed that students make their decisions about ETA use by then (as in Figure 1a). Or, if the minimum valid time is the test start time, it indicates that students decide whether to use the offered ETA at or before the start of the test (see Figure 1b). This minimum time should be determined based on domain knowledge. Indeed, we motivate this assumption based on our NAEP data and prior studies (e.g., Mollenkopf, 1960; Ogut et al., 2025; Weinstein & Roediger, 2012), and we provide further discussion of its plausibility in our setting in Section 7.

Under one-sided noncompliance and stabilized decision, we define the causal estimands of interest and discuss identification and estimation strategies for the target causal estimands.

### 4.1 Estimands

In causal mediation analysis, we decompose the functional total effect (FTE), also referred to as the FATE, into the functional natural direct effect (FNDE) and functional natural indirect effect (FNIE), across the functional domain K . These effects at time *k*, for k∈K , are formally defined as

$$\begin{array}{cccc} \tau_{\text{TE}}(k) & =E\left.l,b,r,a,ceY^{1,T^{1}}(k)-Y^{0,T^{0}}(k),r,b,r,a\right.ce & =⏟{E\left.l,b,r,a,ceY^{1,T^{1}}(k)-Y^{1,T^{0}}(k),r,b,r,a\right.ce}_{=\text{functionalnaturalindirecteffect}}+⏟{E\left.l,b,r,a,ceY^{1,T^{0}}(k)-Y^{0,T^{0}}(k),r,b,r,a\right.ce}_{=\text{functionalnaturaldirecteffect}} & =\tau_{\text{NIE}}(k)+\tau_{\text{NDE}}(k). \end{array}$$

In words, $\tau_{\text{NDE}}(k)$ measures the expected increase in *Y* at time *k* when the treatment changes from Z=0 to Z=1 while holding the mediator constant at the value it would have attained under Z=0 . On the other hand, $\tau_{\text{NIE}}(k)$ measures the expected increase in *Y* at time *k* when the mediator changes to the value it would have attained under Z=0 while holding the treatment constant at Z=1 .^1^ These effects can be written as follows under one-sided noncompliance:

(1) $$\begin{array}{cc} \tau_{\text{NDE}}(k) & =E\left.l,b,r,a,ceY^{1,0}(k)-Y^{0,0}(k),r,b,r,a\right.ce,\\ \tau_{\text{NIE}}(k) & =E\left.l,b,r,a,ceY^{1,T^{1}}(k)-Y^{1,0}(k),r,b,r,a\right.ce. \end{array}$$

For our ETA example, $\tau_{\text{NDE}}(k)$ means the direct effect of receiving ETA at time *k*, not mediated by using it, and $\tau_{\text{NIE}}(k)$ means the indirect effect of receiving ETA through its natural use by a student at time *k*.

Moreover, we are interested in the subgroup effects for the FNDE and FNIE, denoted as S-FNDE and S-FNIE. The S-FNDE and S-FNIE measure the FNDE and FNIE among a subgroup of individuals determined by a subset of pre-treatment covariates *V*, respectively. These subgroup effects examine effect heterogeneity, i.e., how the effects vary across different subgroups. The S-FNDE and S-FNIE are defined as

(3) $$\begin{array}{cc} \tau_{\text{NDE}}(k|V) & =E\left.l,b,r,a,ceY^{1,T^{0}}(k)-Y^{0,T^{0}}(k)|V,r,b,r,a\right.ce=E\left.l,b,r,a,ceY^{1,0}(k)-Y^{0,0}(k)|V,r,b,r,a\right.ce, \end{array}$$

(4) $$\begin{array}{cc} \tau_{\text{NIE}}(k|V) & =E\left.l,b,r,a,ceY^{1,T^{1}}(k)-Y^{1,T^{0}}(k)|V,r,b,r,a\right.ce=E\left.l,b,r,a,ceY^{1,T^{1}}(k)-Y^{1,0}(k)|V,r,b,r,a\right.ce. \end{array}$$

For our ETA example, *V* can be selected as students’ disability status, where V=1 indicates students with disabilities and V=0 indicates those without disabilities. Then, $\tau_{\text{NDE}}(k|V=1)$ is the direct effect of receiving the ETA at time *k* among students with disabilities, while $\tau_{\text{NIE}}(k|V=1)$ is the indirect effect of receiving the ETA via its natural use at time *k* among students with disabilities. Similarly, $\tau_{\text{NDE}}(k|V=0)$ and $\tau_{\text{NIE}}(k|V=0)$ are the corresponding direct and indirect effects, respectively, among students without disabilities.

### 4.2 Identification

The potential variable $T^{z}(k)$ (which equals $T^{z}$ under Assumption A1), i.e., potential ETA use, is inherently derived from a potential functional mediator at time *k* for subjects with Z=z , denoted as $H^{z}(k)$ . Here, $H^{z}(k)$ represents the amount of time a subject would intend to spend on the test at time *k* if they received Z=z . This variable determines the potential intended ETA use variable at time *k*, specifically, 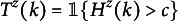, where *c* is the standard testing time. Although $H^{z}(k)$ is only partially observable through *H*, we introduce the variable to identify the FNDE and FNIE within a functional framework (see Appendix S1 of the Supplementary Material for details on the identification and use of $H^{z}(k)$ ). In this article, we focus on the setting of Figure 1a with post-treatment covariates *W*, due to its added complexities compared to the simpler case of Figure 1b with W={} . In the presence of post-treatment covariates, we make the following assumptions to identify the FNDE and FNIE, as well as their subgroup effects (S-FNDE and S-FNIE).

Assumption A2 **(Consistency).**

- If Z=z and $k_{0}\leq k$ , then $T=T^{z}(k)$ and 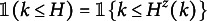.
- If Z=z and T=t and k≤H , then $Y(k)=Y^{z,t}(k)$ .
- If Z=z , then $W=W^{z}$ .

Assumption A3 **(Unconfoundedness).**

For all z∈{0,1} :

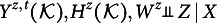
;

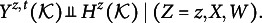

Assumption A4 **(Positivity).**

-
$P_{Z|X}(z|x)>0$ for all z,x .
-
$P_{HW|Z,X}(h,w|z=1,x)>0$ for all x,w and h∈K .
-
$P_{HW|Z,X}(h,w|z=0,x)>0$ for all x,w and $h\in k_{0},c$ .

Assumptions A2–A4 extend the standard identifying assumptions for mediation analysis (Pearl, 2001) to the context of functional outcomes with post-treatment covariates in digital assessments. At a high level, Assumption A2 (Consistency) links observed variables to potential outcomes and mediators; Assumption A3 (Unconfoundedness) states that there are no unmeasured confounders in treatment and mediator assignments, and Assumption A4 (Positivity) ensures sufficient overlap in the distributions of treatment, mediator, and post-treatment covariates between treated units and control units. Note that when post-treatment covariates are absent, one can simply omit or revise parts of the assumptions that include *W* and $W^{z}$ . In Appendix S2 of the Supplementary Material, we provide a set of identifying assumptions for this simplified setting.

More specifically, Assumption A2 states that for A2-(i), a subject’s potential ETA use under a given treatment at time *k*, i.e., $T^{z}(k)$ , is identical to their observed mediator for that same treatment *T*; we remark that $T=T^{Z}=T^{Z}(k)$ for $k_{0}\leq k$ under Assumptions A1 and A2. It also assumes that the indicator of whether $H^{z}(k)$ (i.e., the potential response time at *k*) exceeds or equals time *k* is identical to whether *H* (i.e., the observed total response time) is larger than or equal to *k*.^2^ Assumption A2-(ii) states that when time *k* is less than or equal to *H* (implying that a subject’s data is collected at time *k*), their potential outcome $Y^{z,t}(k)$ is the same as the observed outcome Y(k) if their treatment and mediator were set to Z=z and T=t . Likewise, Assumption A2-(iii) states that their potential post-treatment covariate $W^{z}$ under Z=z is the same as the observed post-treatment covariate *W*.

Assumption A3-(i) states that within every value of the pre-treatment covariates *X*, the treatment *Z* is randomly assigned to subjects, and thus, independent of the potential outcome process $Y^{z,t}(K)$ , the potential functional mediator process $H^{z}(K)$ , and the potential post-treatment covariates $W^{z}$ . In our ETA context, suppose two students have the same pre-treatment covariates, including disability status, English-language learner status, prior math level, and perseverance level. Assumption A3-(i) means that, given these covariates, the ETA receipt status is as good as randomly assigned. Next, Assumption A3-(ii) states that conditional on treatment and the pre- and post-treatment covariates, $H^{z}(K)$ is independent of $Y^{z,t}(K)$ . In particular, it is important to adjust for the post-treatment covariates as well to ensure the independence between $H^{z}(K)$ and $Y^{z,t}(K)$ . To illustrate, consider two students who receive the same treatment (say, Z=1 ) and share identical pre-treatment covariates (e.g., disability status and prior math level) and post-treatment covariates (e.g., items accessed up to $k_{0}$ ). Assumption A3-(ii) implies that given this information, their potential use of ETA (as determined by their potential total response time) is independent of their potential evolving test scores, and thus, the assignment of the mediator (i.e., the ETA use) is as good as random.

Assumption A4 states that (i) for every value of the covariates, the probability of receiving treatment (i.e., the propensity score) is between 0 and 1, (ii) for every value of the pre- and post-treatment covariates, the density distribution of the observed response time *H* over K is non-zero among subjects with Z=1 , and (iii) for every value of the pre- and post-treatment covariates, the density distribution of *H* over $k_{0},c$ is non-zero among subjects with Z=0 . In our ETA example, Assumption A4-(i) means that each eligible student has a real chance of receiving ETA given their pre-treatment covariates, and A4-(ii) and A4-(iii) mean that for each combination of pre-treatment covariates and ETA receipt status, there are students who exhibit all possible values of response time and early-test covariates.

In addition to Assumptions A1–A4, we assume that $Y^{z,t=0}(k)=Y^{z,t=0}(c)$ for k≥c to reflect that a student’s outcome does not change over the extended time period $c,k_{m}$ if they do not use the ETA. In Appendix S1 of the Supplementary Material, we provide formal proofs of identification for the FNDE and FNIE based on these assumptions, building on identification results of Pearl (2001) and Robins (1986). Briefly, the identifying formulas for the FNDE and FNIE depend on the outcome model and the model for the post-treatment variables (i.e., the mediator and the post-treatment confounder), which are defined as

(5) $$\begin{array}{cc} & m_{zt}(k,w,x)=E\{Y^{z,t}(k)|Z=z,W=w,X=x\},\;k\in K \end{array}$$

(6) $$\begin{array}{cc} & g_{zt}(w,x)=P\{T=t,W=w|Z=z,X=x\}, \end{array}$$

where (z,t)∈{(0,0),(1,0),(1,1)} . Note that (z,t)=(0,1) is not considered due to one-sided noncompliance. In Appendix S3 of the Supplementary Material, we also outline the identifying assumptions for the general case with a binary static mediator so that readers can adapt them in their specific contexts.

### 4.3 Estimation

To estimate the FNDE and FNIE, as well as their subgroup effects, we use function-on-scalar regression from the functional data analysis literature (Crainiceanu et al., 2024). Let $S=\{W,X\}\in R^{p+q}$ . We estimate the outcome model (5), i.e., $m_{zt}(k,w,x)$ , using a GAM in the following additive form:

(7) $$\begin{array}{cc} m_{zt}(k,w,x) & =m_{zt}(k,s)=\beta_{0zt}(k)+\sum_{r=1}^{R}f_{rzt}(k,s_{r}). \end{array}$$

In this model, $\beta_{0zt}(k)$ represents the functional intercept, while $f_{rzt}(k,s_{r})$ is its *r*-th smooth function that depends on a subset of covariates $S_{r}\subseteq S$ ( r=1,⋯,R ). These smooth functions can vary across covariate domains and the functional domain and may include time-invariant linear terms, linear interaction terms, as well as one-dimensional and multi-dimensional smooth terms.

As an example, consider the following instance of GAMs for the outcome model:

(8) $$\begin{array}{cc} m_{zt}(k,s) & =\beta_{0zt}(k)+\sum_{j=1}^{p+q}f_{jzt}(s_{j})+\sum_{j=1}^{p+q}f_{jzt}(k,s_{j}). \end{array}$$

Here, $f_{jzt}(s_{j})$ is a time-invariant, univariate smooth function, and $f_{jzt}(k,s_{j})$ is a time-varying smooth function. Both are defined for subjects with Z=z and T=t and for each covariate $S_{j}$ ( j=1,⋯,p+q ).

These smooth functions are parameterized using spline basis expansions. Specifically, for the smooth functions in the outcome model (8), we may take

(9) $$\begin{array}{cc} \beta_{0zt}(k) & =\sum_{l=1}^{L_{0jzt}}\beta_{0zt,l}\phi_{zt,l}(k), \end{array}$$

(10) $$\begin{array}{cc} f_{jzt}(s_{j}) & =\sum_{l=1}^{L_{1jzt}}\beta_{1jzt,l}\phi_{zt,l}(s_{j}), \end{array}$$

(11) $$\begin{array}{cc} f_{jzt}(k,s_{j}) & =\sum_{l=1}^{L_{2jzt}}\beta_{2jzt,l}\phi_{zt,l}(k,s_{j}), \end{array}$$

where $\left\{\phi_{zt,l}(\cdot )\right\}$ are basis functions, and $\left\{\beta_{(\cdot )jzt,l}\right\}$ are the corresponding coefficients. Various classes of basis functions can be used, including B-splines, natural cubic splines, and thin plate splines. In this study, we use thin plate splines. For an overview of thin plate splines, see Section 5.5.1 of Wood (2017) and Wood (2003). We select the smoothing parameter, which controls the trade-off between smoothness and model fit, using the generalized cross-validation (GCV) method. Additionally, we estimate the post-treatment variable model (6) using a similar approach, but with a link function (e.g., logit). More details on its estimation are provided in Appendix S4 of the Supplementary Material.

Let us denote estimators of $m_{zt}$ and $g_{zt}$ in (5) and (6) as $\hat{m_{zt}}$ and $\hat{g_{zt}}$ , respectively. Using these estimators, we construct estimators for the FNDE and FNIE, and their total effect, FTE, as

(12) $$\begin{array}{cc} \hat{\tau_{\text{NDE}}}(k) & =\frac{1}{n}\sum_{i=1}^{n}\sum_{w}\left.l,b,r,a,ce\hat{m_{10}}(k,w,X_{i})\hat{g_{1\cdot }}(w,X_{i})-\hat{m_{00}}(k,w,X_{i})\hat{g_{0\cdot }}(w,X_{i}),r,b,r,a\right.ce, \end{array}$$

(13) $$\begin{array}{cc} \hat{\tau_{\text{NIE}}}(k) & =\frac{1}{n}\sum_{i=1}^{n}\sum_{w}\left.l,b,r,a,ce\hat{m_{11}}(k,w,X_{i})-\hat{m_{10}}(k,w,X_{i}),r,b,r,a\right.ce\hat{g_{11}}(w,X_{i}), \end{array}$$

(14) $$\begin{array}{cc} \hat{\tau_{\text{TE}}}(k) & =\hat{\tau_{\text{NDE}}}(k)+\hat{\tau_{\text{NIE}}}(k), \end{array}$$

where $\hat{g_{z\cdot }}(w,x)=\hat{g_{z0}}(w,x)+\hat{g_{z1}}(w,x)$ , which is an estimator for P(W=w|Z=z,X=x) . If *W* is continuous, the summation over *w* can be replaced with an integral. Likewise, the estimators for the subgroup effects of S-FNDE, S-FNIE, and S-FTE are as follows: 
(15)

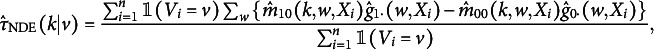



(16)

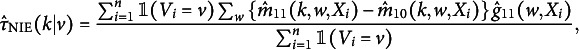

(17) $$\begin{array}{cc} \hat{\tau_{\text{TE}}}(k|v) & =\hat{\tau_{\text{NDE}}}(k|v)+\hat{\tau_{\text{NIE}}}(k|v). \end{array}$$

For inference, we use nonparametric cluster bootstrapping to obtain standard errors or confidence intervals. Specifically, we resample individuals, rather than time points, to account for unit-specific serial dependence. For each sample, we apply the above GAM estimators, i.e., $\hat{\tau_{\text{NDE}}}(k)$ , $\hat{\tau_{\text{NIE}}}(k)$ , $\hat{\tau_{\text{TE}}}(k)$ , $\hat{\tau_{\text{NDE}}}(k|v)$ , $\hat{\tau_{\text{NIE}}}(k|v)$ , and $\hat{\tau_{\text{TE}}}(k|v)$ , and then compute standard errors or pointwise confidence intervals from the bootstrap distribution (see Appendix S5 of the Supplementary Material for details).

## 5 Simulation study

### 5.1 Designs and evaluation

We conducted a simulation study to investigate the finite-sample performance of the proposed estimators. We varied the number of observations *n* from {100,200,500,1,000} . We set the standard testing time to c=20 , the maximum extended testing time to $k_{m}=40$ , and the minimum value of the functional domain to $k_{0}=6$ . For each subject *i*, we generated two binary covariates $X_{i1}$ and $X_{i2}$ independently from Ber(0.5) , and the ETA receipt status $Z_{i}$ from $\text{Ber}(\text{expit}(0.2(X_{i1}+X_{i2}-1))),$ where expit(v)=\exp(v)/{1+\exp(v)} .

For the outcome, we considered the following two data-generating processes: the linear outcome model and the cubic outcome model. The linear outcome model uses 

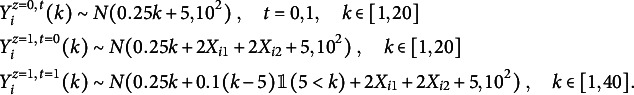

The cubic outcome model uses, for 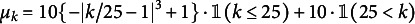 and $\nu_{k}=-10\{-|(k-5)/50-1{|}^{3}+1\}$ , 

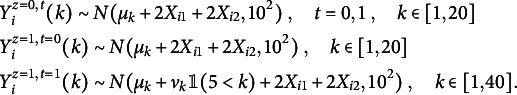

The post-treatment covariate at time $k_{0}=6$ was selected as 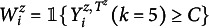, where C=8.25 for the linear outcome model and C=6.88 for the cubic outcome model. Thus, $W_{i}^{z}$ serves as an indicator of whether a student achieved a high test score (or accessed more items) within the first 5 minutes.

We also generated $H_{i}^{z}(k_{0})$ , i.e., the amount of time that subject *i* would intend to spend on the test for $Z_{i}=z$ , conditional on the pre-treatment covariates $\left(X_{i1},X_{i2}\right)$ and the post-treatment covariates $W_{i}^{z}$ , as follows for z=0,1 :

P{Hiz(k0)=(10,15,20)⋅(z+1)}=\lbrace(0.2,0.2,0.6)\textif_Xi1+_Xi2=0\textand_^Wi0=0(0.25,0.25,0.5)\textif_Xi1+_Xi2=0\textand_^Wi0=1(0.3,0.3,0.4)\textif_Xi1+_Xi2=1\textand_^Wi0=0(1/3,1/3,1/3)\textif_Xi1+_Xi2=1\textand_^Wi0=1(1/3,1/3,1/3)\textif_Xi1+_Xi2=2.

Roughly speaking, students with higher values of the covariate sum $X_{i1}+X_{i2}$ and $W_{i}^{z}=1$ (i.e., higher early-stage test scores) will likely have shorter actual response times. Given $H_{i}^{z}(k_{0})$ , we defined $H_{i}^{z}(k)=H_{i}^{z}(k_{0})$ for all k∈K . The ETA use variable at time *k* was determined by 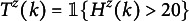. Note that $T^{0}(k)=0$ due to one-sided noncompliance in ETA settings. The observed data for subject *i* were defined by $\left\{X_{i1},X_{i2},Z_{i},W_{i},H_{i},T_{i},Y_{i}(k)\right\}$ for $k\in 6,H_{i}$ , where $\left\{H_{i},T_{i},Y_{i}(k)\right\}$ were obtained from $H_{i}=H_{i}^{Z_{i}}(k)$ , $T_{i}=T_{i}^{Z_{i}}(k)$ , and $Y_{i}(k)=Y_{i}^{Z_{i},T_{i}}(k)$ .

Using the simulated data, we estimated the FNDE, FNIE, and FTE using the proposed GAM estimators in Section 4.3. We denote these estimators as $\hat{\tau_{\text{NDE},GAM}}$ , $\hat{\tau_{\text{NIE},GAM}}$ , and $\hat{\tau_{\text{TE},GAM}}$ . For implementation, we used the function gam from R package mgcv (Wood, 2023) to fit the GAMs for both the outcome and post-treatment variable models. Details on the implementation are provided in Appendix S6 of the Supplementary Material. As comparison estimators, we considered two parametric regression-based estimators for the FNDE, FNIE, and FTE, fitting the smooth curves over time through linear and cubic terms. We refer to them as *linear estimators* (denoted as $\hat{\tau_{\text{NDE},Lin}}$ , $\hat{\tau_{\text{NIE},Lin}}$ , and $\hat{\tau_{\text{TE},Lin}}$ ) and *cubic estimators* (denoted as $\hat{\tau_{\text{NDE},Cub}}$ , $\hat{\tau_{\text{NIE},Cub}}$ , and $\hat{\tau_{\text{TE},Cub}}$ ), respectively (see Appendix S6 of the Supplementary Material for a detailed specification of the coefficients in model (9)). We repeated the simulation 1,000 times.

Based on 1,000 repetitions, we calculated the bias and mean squared error (MSE) of the three estimators, defined as follows for the FNDE:

$$\begin{array}{ccc} \backslash text{Bias}_{\text{NDE},⋆} & =\frac{1}{1000}\sum_{j=1}^{1000}\frac{1}{35}\sum_{k=6}^{40}\left.l,b,r,a,ce\hat{\tau_{\text{NDE},⋆}^{\left(j\right)}}(k)-\tau_{\text{NDE}}(k),r,b,r,a\right.ce\backslash text{MSE}_{\text{NDE},⋆} & =\frac{1}{1000}\sum_{j=1}^{1000}\frac{1}{35}\sum_{k=6}^{40}\left.l,b,r,a,ce\hat{\tau_{\text{NDE},⋆}^{\left(j\right)}}(k)-\tau_{\text{NDE}}(k),r,b,r,a\right.ce^{2}, \end{array}$$

where ⋆∈{GAM,Lin,Cub} denotes the estimator type and the superscript ${}^{\left(j\right)}$ represents the simulation repetition index. The bias and MSE for the FNIE and FTE are similarly defined. Smaller bias and MSE values indicate better performance of an estimator.

We also evaluated the performance of the inferential procedure based on the bootstrap. For each *k*, we calculated the empirical coverage rates of 95% bootstrap confidence intervals for the FNDE based on the proposed GAM method (denoted as $\backslash text{CI}_{\text{NDE},GAM}(k)$ ) as follows: 

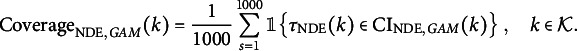

The coverage rates of the empirical coverage rates of 95% bootstrap confidence intervals for the FNIE and FTE are similarly defined. The R code for the simulations is available at https://github.com/qkrcks0218/FDA.

### 5.2 Simulation results

Table 2 summarizes the simulation results under the linear outcome model. In this design, the linear and cubic estimators are correctly specified due to the linear form of the outcome model. However, the cubic estimator includes redundant regressors of higher-order time components. As discussed earlier, the GAM estimator is expected to capture the linear form due to its flexibility and adaptivity. This is confirmed by our finding that all three estimators exhibit negligible bias across all three estimands and sample sizes. In terms of MSE, the linear estimator achieves the smallest value, as it is the correctly specified and parsimonious model. In this design, the linear estimator serves as an oracle estimator. The cubic estimator is less efficient than the linear estimator due to the inclusion of redundant terms. The GAM estimator performs between the linear and cubic estimators, and it consistently shows a smaller MSE than the cubic estimator across all three estimands and sample sizes.

**Table 2:** Simulation results under the linear outcome model Table 2 long description.

*N*	Estimator	FNDE		FNIE		FTE	
		Bias	MSE	Bias	MSE	Bias	MSE
100	GAM	0.052	3.919	− 0.046	1.709	0.009	1.810
	Linear	0.056	2.674	− 0.044	1.324	0.015	1.147
	Cubic	0.016	6.081	− 0.038	2.492	− 0.019	2.841
200	GAM	0.003	1.832	0.004	0.756	0.004	0.906
	Linear	0.000	1.123	− 0.001	0.522	− 0.001	0.556
	Cubic	− 0.010	3.025	0.018	1.210	0.007	1.427
500	GAM	− 0.012	0.637	0.000	0.242	− 0.012	0.327
	Linear	− 0.012	0.441	0.001	0.181	− 0.011	0.224
	Cubic	− 0.013	1.080	− 0.006	0.397	− 0.019	0.531
1,000	GAM	− 0.005	0.340	0.008	0.131	0.003	0.155
	Linear	0.001	0.230	0.003	0.095	0.004	0.104
	Cubic	− 0.016	0.576	0.012	0.214	− 0.004	0.260

*Note*: FNDE = functional natural direct effect; FNIE = functional natural indirect effect; FTE = functional total effect; GAM = generalized additive model; Linear = parametric regression with a linear term; Cubic = parametric regression with linear, quadratic, and cubic terms; MSE = mean squared error.

Next, we summarize simulation results under the cubic outcome model in Table 3. In this design, the cubic estimator is still correctly specified, but the linear estimator is mis-specified. Despite the change in the outcome model form, the GAM estimator is expected to capture the true model with no concerns about mis-specification. In line with this expectation, both the GAM and cubic estimators exhibit negligible bias, and the GAM estimator generally shows slightly larger absolute bias. In contrast, the linear estimator shows substantial bias for all three estimands and sample sizes. Regarding MSE, the GAM estimator generally achieves the smallest value, and it outperforms the cubic estimator. Although the GAM estimator exhibits (slightly) larger bias than the cubic estimator, its smaller MSE highlights a superior bias–variance trade-off. In contrast, the linear estimator yields much larger MSE due to model mis-specification.

**Table 3:** Simulation results under the cubic outcome model Table 3 long description.

*N*	Estimator	FNDE		FNIE		FTE	
		Bias	MSE	Bias	MSE	Bias	MSE
100	GAM	− 0.033	4.063	− 0.057	2.088	− 0.087	2.094
	Linear	− 0.156	2.590	− 0.096	2.407	− 0.247	2.416
	Cubic	0.006	6.171	0.017	2.634	0.030	2.995
200	GAM	− 0.033	2.007	− 0.062	0.877	− 0.096	1.121
	Linear	− 0.141	1.222	− 0.110	1.604	− 0.251	1.799
	Cubic	− 0.022	3.083	0.028	1.231	0.004	1.570
500	GAM	0.015	0.765	− 0.059	0.339	− 0.044	0.459
	Linear	− 0.141	0.471	− 0.112	1.201	− 0.253	1.433
	Cubic	0.008	1.123	0.024	0.448	0.032	0.606
1,000	GAM	0.027	0.404	− 0.069	0.182	− 0.041	0.225
	Linear	− 0.126	0.247	− 0.134	1.113	− 0.260	1.302
	Cubic	0.008	0.525	0.001	0.218	0.009	0.268

*Note*: FNDE = functional natural direct effect; FNIE = functional natural indirect effect; FTE = functional total effect; GAM = generalized additive model; Linear = parametric regression with a linear term; Cubic = parametric regression with linear, quadratic, and cubic terms; MSE = mean squared error.

**Figure 2:**
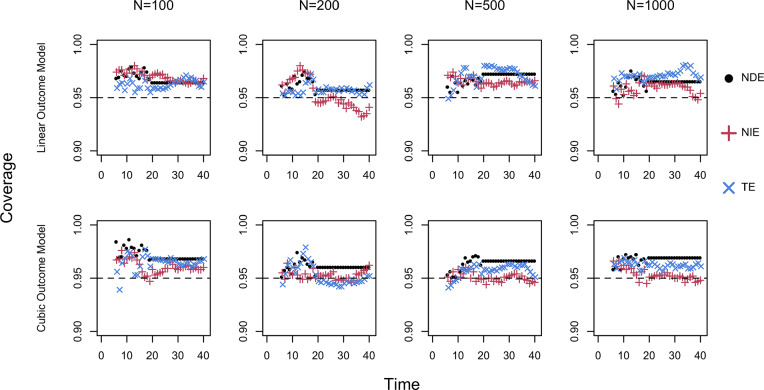
Coverage of 95% bootstrap confidence intervals. The horizontal black line provides a reference for the nominal level 95%; FNDE = functional natural direct effect; FNIE = functional natural indirect effect; FTE = functional total effect. Figure 2 long description.

Figure 2 visually presents the coverage rates under linear and cubic outcome scenarios over the functional domain. In both scenarios, the coverage rates are close to the nominal 95% level across all the estimands and sample sizes. This indicates that the bootstrap procedure provides valid inference.

Overall, our simulation study demonstrates that the GAM estimator performs at least as well as, and often better than, correctly specified parametric models. This emphasizes its capacity to efficiently estimate functional causal effects without concerns about model mis-specification. Additionally, the proposed bootstrap-based method performs well in both outcome models, and it ensures reliable statistical inference with the GAM estimator.

## 6 Empirical example: ETA

### 6.1 Data and variables

In our study, we used the 2017 NAEP Grade 8 restricted-use process data for mathematics to estimate the functional total, direct, and indirect effects of receiving ETA. The 2017 NAEP math assessment was administered with multiple test forms, each consisting of two blocks. We used process data from one block, named M3 , across multiple test forms. Note that test forms were randomly assigned to students, so ignoring form differences should not introduce bias. The selected block contains 15 items, including multiple choice items, selected response items, and constructed response items. The actual items are available on the website: https://www.nationsreportcard.gov/nqt/searchquestions. Within each block, students could revisit and change their answers.

The standard testing duration is 30 minutes, and the extended time adds extra 60 minutes, so the full testing time domain ranges from 1 to 90 minutes. In our data analysis, we set $k_{0}=6$ (i.e., 20% of the standard 30-minute testing time). That is, students’ decisions to use ETA are assumed to stabilize after the first 6 minutes, and thus, their early-test responses up to 6 minutes serve as post-treatment covariates. We select $k_{0}=6$ because this initial 20% of the testing period serves as a reasonable window where students form their impressions and decide whether to use ETA. Importantly, we do not claim that $k_{0}=6$ is universally correct. Rather, it is a context-specific choice based on prior literature and testing situations. Using $k_{0}=6$ , we incorporate a binary post-treatment covariate about whether the number of items accessed by 6 minutes exceeds the sample median. However, if researchers believe that decisions about the use of ETA are made prior to the test, they do not need to include post-treatment covariates, and the setting becomes Figure 1b. Or, if ETA use decisions are believed to stabilize later in the test, say 15 minutes, they may set $k_{0}=15$ and incorporate additional test-taking covariates up to 15 minutes. Additionally, the end of the functional domain $k_{m}$ was 76 minutes because the range (76, 90] has a relatively small number of students. For these reasons, we used the range [6, 76] as our functional domain of interest.

Our target sample consists of students eligible for ETA, i.e., *students with disabilities* and *English language learners*. We excluded students who spent less than 2 minutes on the test due to extremely low efforts, as well as those who spent more than 76 minutes due to small sample sizes at time *k*. As a result, our final analysis sample included 4,130 students^3^ (99.2% of the eligible sample for ETA in the process data), with about 66% of these students having disabilities, and 33% being English language learners without disabilities.

The functional outcomes of interest are test scores and item access (the number of items a student accessed). We constructed these two outcomes by transforming the raw, unstructured process data into a functional format. The first outcome is a student’s evolving test scores on a 15-item math test block over time, and ranges from 0 to 25 points. The second outcome is a student’s evolving item access over time, ranging from 0 to 15 items. In this study, we counted a student’s access to an item only if they spent more than 3 seconds on it because shorter visits are not considered effortful.

In addition, our treatment variable *Z* is whether a student received ETA or not, where Z=1 denotes that a student received ETA and Z=0 denotes that a student did not receive it. Our mediator variable *T* is whether a student used ETA or not, where T=1 denotes that a student used ETA and T=0 denotes that a student did not use it. H is a student’s observed total response time, and when H is larger than 32 minutes, T=1 ; otherwise, T=0 . Following prior works (e.g., Lee & Suk, 2025; Suk & Kim, 2025), we use 32 minutes (rather than 30 minutes) as the end of the standard testing period due to interruptions in the typical testing experiences during the NAEP test. We assume one-sided noncompliance between the treatment *Z* and mediator *T*, where some ETA recipients did not use ETA, but all non-recipients did not use it. As in prior work, only about 30% of the recipients used ETA, and similar patterns were found among both students with disabilities and English language learners.

Furthermore, we used students’ disability status as a moderator *V* to explore subgroup effects. We also used a set of pre-treatment covariates *X*, including students’ sex, race, disability status, limited English proficiency status, free lunch status, parental education, interest in math, and a proxy for the current math course level. These variables partially explained why some eligible students did not receive or use ETA. For a full list of the variables used in data analysis, see Appendix S7 of the Supplementary Material.

Following our proposed methods discussed in Section 4.3, we used GAM estimators to estimate the FNDE, FNIE, and FTE, as well as their subgroup effects. Following the approach used in the simulation study, we employed the gam function from the R package mgcv (Wood, 2023) to fit GAMs for both the outcome and post-treatment variable models. In the gam function, we selected knots=18 and gamma=1.5, which resulted in no diagnostic issue via the gam.check command. See Appendix S6 of the Supplementary Material for details on the implementation. We also conducted nonparametric bootstrapping to estimate confidence intervals for each effect in R software.

As a final note, we did not apply sampling weights and the corresponding jackknife replicate weights provided in the 2017 NAEP data. This allows us to focus on the demonstrations of our methods without introducing additional complexity from the sampling design. However, our results are specific to the analysis sample and should not be generalized to the broader population.

### 6.2 Results

**Figure 3:**
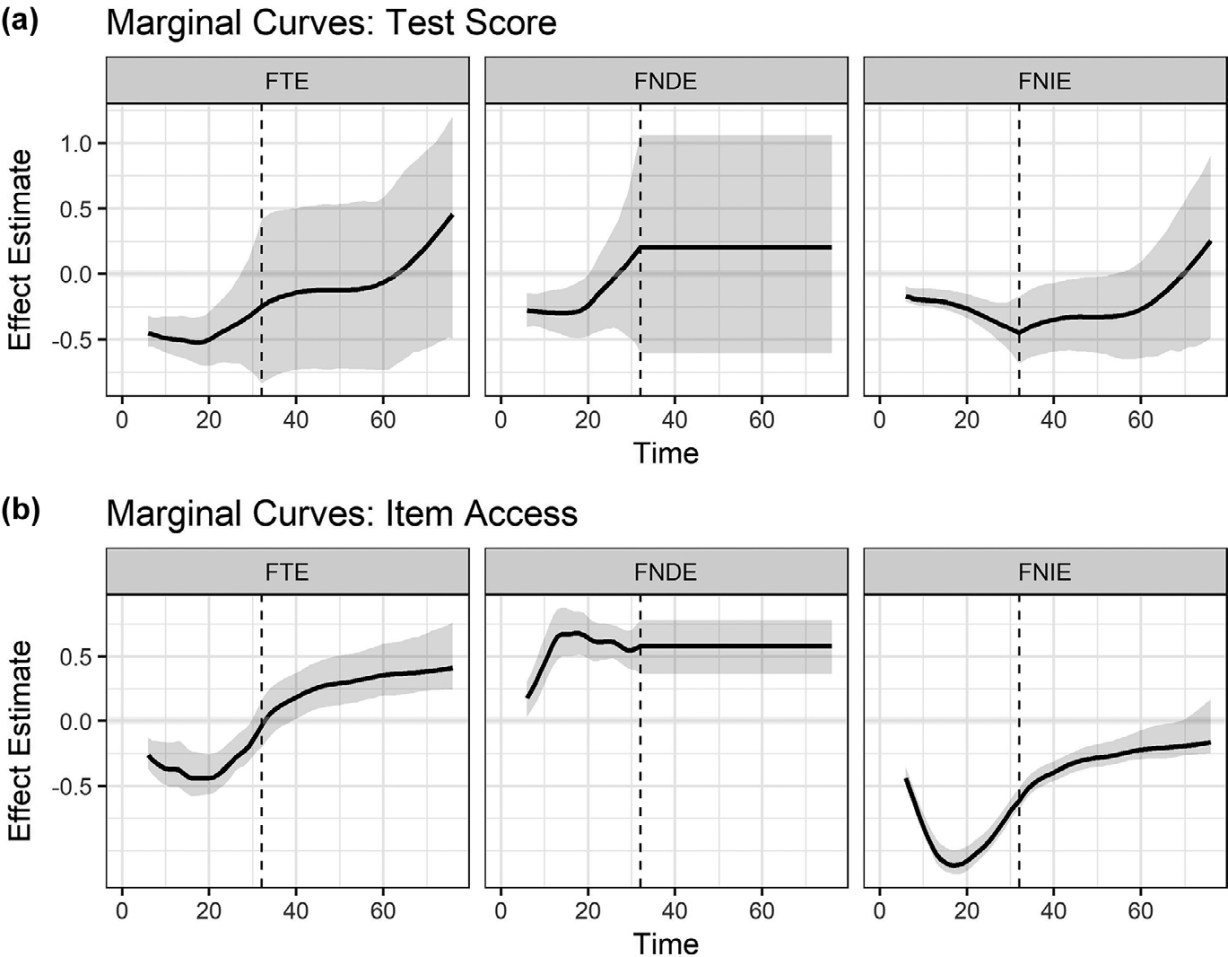
Marginal curves for the functional effects. *Note*: Shaded gray area indicates the 95% confidence bands for each effect, a dashed vertical line indicates the end of standard testing time, and a thick gray horizontal line indicates an effect size of zero. FTE = functional total effect; FNDE = functional natural direct effect; FNIE = functional natural indirect effect. *Source*: U.S. Department of Education, National Center on Educational Statistics (NCES), 2017 NAEP Grade 8 Mathematics Process Data, Student Features Data File Partial Form and Response Data File. Figure 3 long description.

Figure 3 summarizes the estimated FTE, FNDE, and FNIE of receiving ETA on two outcomes—test score and item access—over the functional domain ranging from 6 to 76 minutes. The figure includes the 95% confidence bands (shaded gray area) for each effect over time. A dashed vertical line indicates the end of standard testing time (here, 32 minutes), and a thick gray horizontal line indicates an effect size of zero. From Figure 3a, the estimated FTE curve for test scores shows a non-linear trend over time. Initially, the effect is significantly negative and shows a decreasing pattern until around 19 minutes. However, the effect begins to increase after this point, crosses zero near 60 minutes, and continues to rise toward the end of the time period. This pattern suggests that the FTE on test scores improves over time, and a positive effect emerges during the later phase of the extended time period, although it is not significant. After decomposing the FTE into FNDE and FNIE, the direct effect (FNDE) curve starts with a negative effect, but increases sharply between 20 and 32 minutes, remaining constant afterward. This constant effect is expected because the extended time period is inactive for both ETA non-recipients and non-users. In contrast, the indirect effect (FNIE) curve gradually decreases to about − 0.4 near 32 minutes. Then, it turns upward, becomes positive after 70 minutes, and continues to increase until the end of the testing period. Overall, during the later phase of the extended time period, the marginal total effect of receiving ETA on test scores (FTE) shows a positive and increasing trend. This total effect is initially driven by psychological benefits from receiving ETA on the test scores, and later by the increasing effect of actually using it, although these direct and indirect effects are non-significant throughout the time period.

For item access, shown in Figure 3b, the FTE curve starts with a significantly negative effect and exhibits a decreasing trend until around 18 minutes. After this point, the curve rises rapidly until the end of the standard testing period and then gradually increases during the extended time period. After effect decomposition, the FNDE curve for item access is significantly positive throughout the entire time period, which differs from the results obtained with test scores. It increases rapidly until 15 minutes, slightly decreases by the end of the standard testing period, and then remains constant. This positive direct effect indicates that students who receive ETA but do not use it gain access to items more quickly than those who do not receive ETA. This may be because ETA availability reduces test anxiety or allows students to skim through items initially. In contrast, the FNIE curve declines sharply and reaches its most negative value of − 1.7 near 18 minutes. Then, it rises steadily and reaches a level that is not significantly different from zero. This finding indicates that students who use ETA have a slower rate of item access compared to non-users, but eventually catch up on unaccessed items over the extended time period.

**Figure 4:**
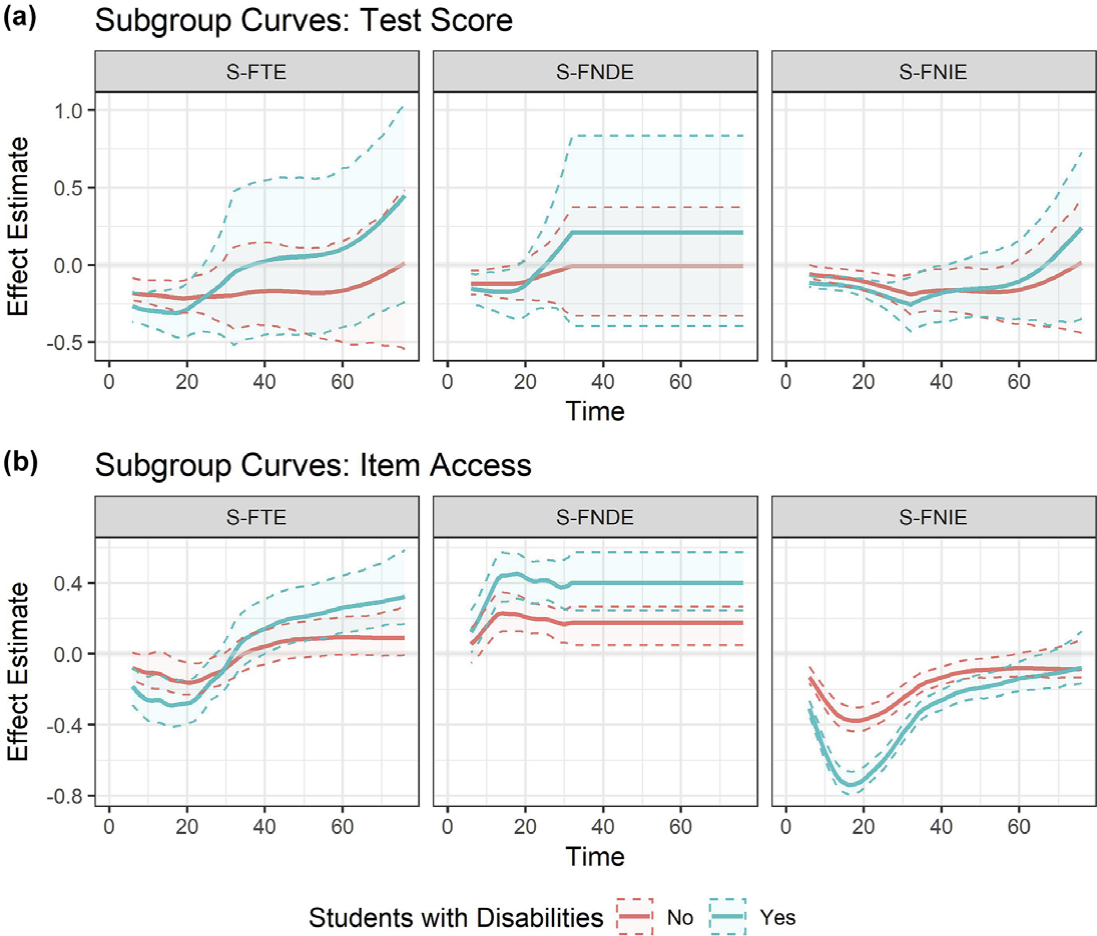
Subgroup curves for the functional effects. *Note*: Dashed lines indicate the 95% confidence bands for each effect, and a thick gray horizontal line indicates an effect size of zero. S-FTE = subgroup functional total effect; S-FNDE = subgroup functional natural direct effect; S-FNIE = subgroup functional natural indirect effect. *Source*: U.S. Department of Education, National Center on Educational Statistics (NCES), 2017 NAEP Grade 8 Mathematics Process Data, Student Features Data File Partial Form and Response Data File. Figure 4 long description.

To explore heterogeneity of the functional effects, we further examine the S-FTE, S-FNDE, and S-FNIE based on students’ disability status (see Figure 4 for the results, where dashed lines represent the 95% confidence bands). For test scores (Figure 4a), students with disabilities (blue curve) show a larger negative effect of FTE compared to those without disabilities (red curve) until 25 minutes. After this point, students with disabilities show a more rapid increase, although the differences in FTE are not significant given the overlapping confidence bands. When focusing on direct and indirect effects, the FNDE is higher for students with disabilities, particularly after 20 minutes, where their curve stabilizes at a higher level compared to those without disabilities. However, these differences are also not significant. In terms of FNIE, students with disabilities show a sharper upward trend after 40 minutes compared to the gradual increase among students without disabilities, but again, the differences are not significant.

For item access (Figure 4b), we observe notable differences between students with and without disabilities. The FTE for item access is more negative for students with disabilities until around 30 minutes, but after this point, the curve becomes positive more quickly and continues to increase until the end. Students without disabilities show a flatter trend after 40 minutes. The differences in FTE between the two groups are not significant. In contrast, we find significant differences in FNDE and FNIE. Specifically, the FNDE curve for students with disabilities rises more sharply and stabilizes at a significantly higher level compared to those without disabilities. This finding indicates that the psychological effect of receiving ETA on item access is significantly greater among students with disabilities. For the FNIE, students with disabilities show a steeper decline initially and show significantly lower effects compared to those without disabilities. However, this negative effect recovers and after around 50 minutes, there are no significant differences between the two groups. This result suggests that although students with disabilities may initially access items at a slower pace, they eventually catch up on unaccessed items within the extended time period.

## 7 Discussion and conclusions

This article extends the standard causal mediation framework to the context of functional outcomes to identify and estimate the FNDE, FNIE, and FTE, as well as their subgroup effects, using process data from digital assessments. Our framework enables researchers to answer the multi-faceted causal question: “*Why, when, and for whom does a program work?*”. Within this framework, we provide the counterfactual definitions of these effects and present nonparametric identification strategies for each effect depending on the presence or absence of post-treatment covariates. To estimate these effects, we construct the GAM-based estimators using function-on-scalar outcome regression and compute their standard errors and confidence intervals via nonparametric bootstrapping. Our simulation study reveals that the GAM estimators perform as well as, and often better than correctly specified parametric methods, with no concerns about model mis-specification. The proposed bootstrap-based method ensures reliable statistical inference with the GAM estimators. Furthermore, we demonstrate our proposed methods in a real-world application on the ETA using NAEP process data. In this analysis, we estimate the FTE, FNDE, and FNIE of receiving ETA for two outcomes—test scores and item access—and also examine their subgroup effects depending on students’ disability status. This application uncovers the dynamic causal mechanisms underlying the effects of ETA on functional outcomes and highlights when and for whom each effect works during the testing period.

In our framework, Assumption A1 (stabilized decision) means that students form a plan regarding ETA use before or early in the test. This assumption is crucial for defining the functional effects of interest, but it contrasts with the alternative view that the decision to use ETA evolves continuously until the end of the standard testing time. Empirical evidence supports the stabilized view. In our 2017 NAEP data, more than 25% of the students without disabilities and without ETA experienced time pressure toward the end of the test (Ogut et al., 2025). This suggests a strong need for extra time among our target sample (i.e., students with disabilities and English language learners), but only about 30% of ETA recipients actually used it. This low rate is difficult to explain via a continuously evolving decision process, but aligns well with Assumption A1. In addition, prior research on testing behavior supports the plausibility of Assumption A1; students form rapid global impressions of a test’s difficulty based on the earliest items (e.g., Anaya et al., 2022; Jackson & Greene, 2014; Weinstein & Roediger, 2012), and they plan test-taking strategies in advance when facing time limits (Mollenkopf, 1960). These findings imply key strategic decisions, including ETA use, are likely determined before or during the early phase of the test.

Moreover, our proposed approach is timely and highly relevant in today’s data-rich environment. As digital devices have become widely used, vast amounts of functional data are being generated across various fields. While this rich information is undoubtedly valuable, conventional methods may not be able to handle such complex data. Our approach addresses this challenge by developing a novel causal tool, specifically designed for functional data. While existing causal mediation and moderation methods can reveal the causal mechanisms within subgroups, these methods are still limited in answering crucial questions, such as when or where a program is effective. Our proposed approach extends these existing methods by investigating the causal effects over the functional domain of interest (e.g., time and space) to help uncover more nuanced and detailed causal evidence from complex data. Furthermore, our study tackles another challenge of analyzing time-stamped process data by demonstrating how to generate functional outcomes from such data and examine them with functional data analysis methods. We focus on function-on-scalar regression because of its suitability for functional outcomes, but other functional data methods hold great promise in further advancing the analysis of complex process data from computer-based testing and learning environments.

Based on the findings of this article, we provide limitations of this article and some suggestions for future research. First, in Assumption A1 in Section 4, we assume that test-takers’ decisions to use ETA stabilize at a certain time $k_{0}$ (e.g., at the start, within the first 20% of the standard testing time). However, in some testing contexts (e.g., high-stakes or highly speeded assessments), the decision to use ETA may not stabilize but instead evolve continuously. To the best of our knowledge, such dynamic decision-making has not been systematically examined, but future research would investigate the stability of these decisions across different testing conditions. Second, if researchers hypothesize a continuously evolving decision regarding ETA use, they should model the dynamic interactions among item access, test scores, and test drop-out (i.e., whether a test-taker exits the test) at each time point. This would require a survival analysis framework that treats test drop-out as a censoring event. In this framework, ETA use should be modeled as a functional mediator rather than a static mediator. Future work would investigate how to extend our framework to the context of functional mediators.

Third, we assume that all the confounders are measured in the observed data. Since observed data may suffer from potential unmeasured confounders, future research would develop sensitivity analysis tools or robust causal methods against unmeasured confounding, for example, by extending proximal causal inference approaches (Tchetgen Tchetgen et al., 2024). Fourth, our estimation methods focus on outcome regression, weighted by quantities derived from the post-treatment confounder model. In our setting, it is possible to develop more robust estimators that satisfy double robustness. Such methods would be consistent if either the treatment assignment (propensity score) model or the outcome model is consistently estimated, thereby increasing robustness to model mis-specification.

Overall, although our approach has some limitations, we believe that our proposed framework and methods contribute to a more nuanced understanding of dynamic causal mechanisms in functional data, ultimately advancing program evaluation efforts in the social sciences.

## Supporting information

10.1017/psy.2026.10087.sm001 Suk and Park supplementary material Suk and Park supplementary material

## Data Availability

This article used the restricted-use version of the Grade 8 Mathematics Process Data from the 2017 National Assessment of Educational Progress (NAEP). A restricted-use data license can be obtained through the IES Electronic Application System (https://nces.ed.gov/statprog/instruct.asp).

## Footnotes

1 We could decompose the total effect into $\tau_{\text{NDE}}^{*}(k)=E\left.l,b,r,a,ceY^{1,T^{1}}(k)-Y^{0,T^{1}}(k),r,b,r,a\right.ce$ (i.e., direct effect) and $\tau_{\text{NIE}}^{*}(k)=E\left.l,b,r,a,ceY^{0,T^{1}}(k)-Y^{0,T^{0}}(k),r,b,r,a\right.ce$ (i.e., indirect effect). However, in our context, $Y^{0,1}(k)$ is practically not meaningful due to one-sided noncompliance, so we focus on the estimands $\tau_{\text{NDE}}(k)$ and $\tau_{\text{NIE}}(k)$ .

2 The assumption  would hold if we make a stronger assumption $H^{z}(k)=H$ . But we do not assume $H^{z}(k)=H$ because $H^{z}(k)$ is a time-varying quantity, and it is unrealistic to assume that it is identical to the observed variable *H* over the entire functional domain. Therefore, we make a weaker and more realistic assumption about the binary relationship between *k* and $H^{z}(k)$ , which is also sufficient for causal identification.

3 Numbers are rounded to the nearest ten in accordance with NAEP’s public reporting policy.

## Supplementary material

The supplementary material for this article can be found at https://doi.org/10.1017/psy.2026.10087.

## Table 1 Long description

The table has seven columns, ordered left to right: i, X equals open brace X sub 1, X sub 2, X sub 3 close brace, Z, T, H, W, and Y of open brace 3, 4, up to H close brace. The first data row has i equals 1, X equals open brace 1, 0, 0 close brace, Z equals 0, T equals 0, H equals 5, W equals 2, Y equals open brace 1, 2, 3 close brace. The second row has i equals 2, X equals open brace 0, 1, 1 close brace, Z equals 1, T equals 0, H equals 4, W equals 1, Y equals open brace 1, 2 close brace. The next row uses vertical ellipses in all columns to indicate continuation. The last row has i equals n, X equals open brace 1, 1, 1 close brace, Z equals 1, T equals 1, H equals 7, W equals 1, Y equals open brace 0, 1, 2, 2, 3 close brace.

Navigate back to Table 1.

## Figure 1 Long description

The left panel shows five nodes labeled X, W, Z, T, and Y. X points to W, Z, T, and Y. W points to T and Y. Z points to T and Y. T points to Y. There is a curved arrow labeled D from T to Y and a straight arrow labeled I from T to Y. The right panel shows four nodes labeled X, Z, T, and Y. X points to Z, T, and Y. Z points to T and Y. T points to Y. There is a curved arrow labeled D from T to Y and a straight arrow labeled I from T to Y. The left panel is titled Post-treatment covariate W, the right panel is titled Pre-treatment covariate X only.

Navigate back to Figure 1.

## Table 2 Long description

The table is organized by sample size N in four blocks: 100, 200, 500, and 1,000. For each N, three estimators are listed in order: G A M, Linear, and Cubic. Each estimator row contains values for FNDE, FNIE, and FTE, each with Bias and M S E columns. For N equals 100: G A M has FNDE Bias 0.052, M S E 3.919; FNIE Bias negative 0.046, M S E 1.709; FTE Bias 0.009, M S E 1.810. Linear has FNDE Bias 0.056, M S E 2.674; FNIE Bias negative 0.044, M S E 1.324; FTE Bias 0.015, M S E 1.147. Cubic has FNDE Bias 0.016, M S E 6.081; FNIE Bias negative 0.038, M S E 2.492; FTE Bias negative 0.019, M S E 2.841. For N equals 200: G A M has FNDE Bias 0.003, M S E 1.832; FNIE Bias 0.004, M S E 0.756; FTE Bias 0.004, M S E 0.906. Linear has FNDE Bias 0.000, M S E 1.123; FNIE Bias negative 0.001, M S E 0.522; FTE Bias negative 0.001, M S E 0.556. Cubic has FNDE Bias negative 0.010, M S E 3.025; FNIE Bias 0.018, M S E 1.210; FTE Bias 0.007, M S E 1.427. For N equals 500: G A M has FNDE Bias negative 0.012, M S E 0.637; FNIE Bias 0.000, M S E 0.242; FTE Bias negative 0.012, M S E 0.327. Linear has FNDE Bias negative 0.012, M S E 0.441; FNIE Bias 0.001, M S E 0.181; FTE Bias negative 0.011, M S E 0.224. Cubic has FNDE Bias negative 0.013, M S E 1.080; FNIE Bias negative 0.006, M S E 0.397; FTE Bias negative 0.019, M S E 0.531. For N equals 1,000: G A M has FNDE Bias negative 0.005, M S E 0.340; FNIE Bias 0.008, M S E 0.131; FTE Bias 0.003, M S E 0.155. Linear has FNDE Bias 0.001, M S E 0.230; FNIE Bias 0.003, M S E 0.095; FTE Bias 0.004, M S E 0.104. Cubic has FNDE Bias negative 0.016, M S E 0.576; FNIE Bias 0.012, M S E 0.214; FTE Bias negative 0.004, M S E 0.260. Negative values are indicated by the word ‘negative’ before the number. FNDE is functional natural direct effect, FNIE is functional natural indirect effect, FTE is functional total effect, G A M is generalized additive model, M S E is mean squared error.

Navigate back to Table 2.

## Table 3 Long description

The table presents simulation results for sample sizes N equal to 100, 200, 500, and 1,000. For each N, three estimators are compared: GAM, Linear, and Cubic. Columns are grouped by effect type: FNDE, FNIE, and FTE, each with Bias and MSE. For N equals 100: GAM shows FNDE Bias negative 0.033, MSE 4.063; FNIE Bias negative 0.057, MSE 2.088; FTE Bias negative 0.087, MSE 2.094. Linear estimator: FNDE Bias negative 0.156, MSE 2.590; FNIE Bias negative 0.096, MSE 2.407; FTE Bias negative 0.247, MSE 2.416. Cubic estimator: FNDE Bias 0.006, MSE 6.171; FNIE Bias 0.017, MSE 2.634; FTE Bias 0.030, MSE 2.995. For N equals 200: GAM FNDE Bias negative 0.033, MSE 2.007; FNIE Bias negative 0.062, MSE 0.877; FTE Bias negative 0.096, MSE 1.121. Linear: FNDE Bias negative 0.141, MSE 1.222; FNIE Bias negative 0.110, MSE 1.604; FTE Bias negative 0.251, MSE 1.799. Cubic: FNDE Bias negative 0.022, MSE 3.083; FNIE Bias 0.028, MSE 1.231; FTE Bias 0.004, MSE 1.570. For N equals 500: GAM FNDE Bias 0.015, MSE 0.765; FNIE Bias negative 0.059, MSE 0.339; FTE Bias negative 0.044, MSE 0.459. Linear: FNDE Bias negative 0.141, MSE 0.471; FNIE Bias negative 0.112, MSE 1.201; FTE Bias negative 0.253, MSE 1.433. Cubic: FNDE Bias 0.008, MSE 1.123; FNIE Bias 0.024, MSE 0.448; FTE Bias 0.032, MSE 0.606. For N equals 1,000: GAM FNDE Bias 0.027, MSE 0.404; FNIE Bias negative 0.069, MSE 0.182; FTE Bias negative 0.041, MSE 0.225. Linear: FNDE Bias negative 0.126, MSE 0.247; FNIE Bias negative 0.134, MSE 1.113; FTE Bias negative 0.260, MSE 1.302. Cubic: FNDE Bias 0.008, MSE 0.525; FNIE Bias 0.001, MSE 0.218; FTE Bias 0.009, MSE 0.268. Across all sample sizes, the Cubic estimator generally shows the lowest bias for FNDE and FTE, while GAM and Linear estimators have higher bias, especially at smaller N. MSE decreases as N increases for all estimators. FNDE, FNIE, and FTE stand for functional natural direct, indirect, and total effects, respectively. GAM is generalized additive model.

Navigate back to Table 3.

## Figure 2 Long description

Panels are organized in two rows and four columns. The top row is labeled Linear Outcome Model, the bottom row Cubic Outcome Model. Columns are labeled N equals 100, N equals 200, N equals 500, and N equals 1000. Each panel plots coverage on the y axis from 0.90 to 1.00 and time on the x axis from 0 to 40. Three effect types are shown: F N D E as black dots, F N I E as red plus signs, and F T E as blue Xs. A horizontal dashed line marks 0.95 coverage. In all panels, F N D E values cluster near or above 0.95, F N I E values fluctuate around or below 0.95, and F T E values are more variable but generally above 0.95. As sample size increases from left to right, coverage variability decreases for all effects. The cubic outcome model (bottom row) shows more fluctuation in F N I E and F T E at smaller sample sizes compared to the linear model. The legend at right identifies black dots as N D E, red plus as N I E, and blue X as T E.

Navigate back to Figure 2.

## Figure 3 Long description

Top row, labeled Marginal Curves: Test Score, contains three panels. Each panel has x-axis labeled Time (0 to 70) and y-axis labeled Effect Estimate (from -0.5 to 1.0). The first panel, F T E, shows a line starting below zero, rising gradually after the dashed vertical line at 35, and ending above zero. The second panel, F N D E, shows a line starting below zero, rising sharply at the dashed line, and flattening above zero. The third panel, F N I E, shows a line starting near zero, dipping below zero before the dashed line, then rising above zero. All panels have a thick gray horizontal line at zero and a shaded gray area indicating the 95 percent confidence band. Bottom row, labeled Marginal Curves: Item Access, also contains three panels with the same axes. The first panel, F T E, shows a line starting below zero, dipping further, then rising above zero after the dashed line. The second panel, F N D E, shows a line rising sharply to above zero at the dashed line, then flattening. The third panel, F N I E, shows a line starting above zero, dipping sharply below zero before the dashed line, then rising above zero. All panels include the same horizontal and shaded features as the top row.

Navigate back to Figure 3.

## Figure 4 Long description

The top panel labeled Subgroup Curves: Test Score contains three graphs from left to right: S F T E, S F N D E, and S F N I E. Each graph plots effect estimate on the y-axis and time on the x-axis. The thick gray horizontal line marks zero effect size. Two sets of curves are shown: red dashed lines for students without disabilities and blue dashed lines for students with disabilities, each with solid lines for the main effect and dashed lines for the 95 percent confidence bands. In S F T E and S F N D E, the blue curves rise above zero after time equals 40, while red curves remain near zero. In S F N I E, both curves increase after time equals 60, with blue curves rising more sharply. The bottom panel labeled Subgroup Curves: Item Access also contains S F T E, S F N D E, and S F N I E graphs. Blue curves for students with disabilities show higher effect estimates after time equals 40 in S F T E and S F N D E, while S F N I E curves for both groups dip below zero before rising after time equals 40. The legend below indicates red dashed lines represent ‘No’ and blue dashed lines represent ‘Yes’ for students with disabilities.

Navigate back to Figure 4.
