# Supplementary material for: Causal Mediation and Functional Outcome Analysis with Process Data
Source: Psychometrika. 2026 Jan 23;91(2):424–44. doi: 10.1017/psy.2026.10087 (PMC13294617; doi:10.1017/psy.2026.10087)
Supplement: Suk and Park supplementary material [file S0033312326100878sup001.pdf]

Supplementary Materials

S1 Identification for the FNDE and FNIE

First, we establish identification of $E\{Y^{0,T^0}(k) \,|\, X\}$ as follows:

- (Case 1) For $k \in [k_0, c]$:

$$
\begin{aligned}
&E\Big\{Y^{0,T^0}(k)\,\Big|\,X\Big\} \\
&= E\Big\{Y^{0,0}(k)\,\Big|\,X\Big\} && \text{One-sided noncompliance}\\
&= E\Big\{Y^{0,0}(k)\,\Big|\,Z=0,X\Big\} && \text{Assumption A3-(i)}\\
&= E[E\Big\{Y^{0,0}(k)\,\Big|\,Z=0,W,X\Big\}\,\Big|\,Z=0,X\Big] && \text{Law of iterated expectation}\\
&= E\Big[E\Big\{Y^{0,0}(k)\,\Big|\,H^0(k)\in[k,k_m],Z=0,W,X\Big\}\,\Big|\,Z=0,X\Big] && \text{Assumption A3-(ii)}\\
&= E\Big[E\Big\{Y^{0,0}(k)\,\Big|\,H\in[k,k_m],Z=0,W,X\Big\}\,\Big|\,Z=0,X\Big] && \text{Assumption A2-(i)}\\
&\overset{(*)}{=} E\Big[E\Big\{Y^{0,0}(k)\,\Big|\,H\in[k,c],Z=0,W,X\Big\}\,\Big|\,Z=0,X\Big] && \\
&= E\Big[E\Big\{Y(k)\,\Big|\,H\in[k,c],Z=0,W,X\Big\}\,\Big|\,Z=0,X\Big] && \text{Assumption A2-(ii)}
\end{aligned}
$$

Note that equality $\overset{(*)}{=}$ holds from the observation that the event $\{H \in [k, k_m]\} \cap \{Z = 0\}$ is the same as the event $\{H \in [k, c]\} \cap \{Z = 0\}$.

- (Case 2) For $k \in (c, k_m]$: We have $Y^{0,0}(k) = Y^{0,0}(c)$. Therefore,

$$
E\Big\{Y^{0,T^0}(k)\,\Big|\,X\Big\} = E\Big\{Y^{0,T^0}(c)\,\Big|\,X\Big\} = E\Big[E\Big\{Y(k)\,\Big|\,H=c,Z=0,W,X\Big\}\,\Big|\,Z=0,X\Big]
$$

Combining (Case 1) and (Case 2), we have the following result for $k \in [k_0, k_m]$:

$$
E\Big\{Y^{0,T^0}(k)\,\Big|\,X\Big\} = E\Big[E\Big\{Y(k\wedge c)\,\Big|\,H\in[k\wedge c,c],Z=0,W,X\Big\}\,\Big|\,Z=0,X\Big]\,. \tag{S1}
$$

Here, $a \wedge b = \min(a, b)$.

Second, we establish identification of $E\{Y^{1,T^0}(k) \,|\, X\}$ as follows:

- (Case 1) For $k \in [k_0, c]$: From the mediation formula of Pearl (2001), we find the following result for $k \in [k_0, c]$:

$$
\begin{aligned}
& E\Big\{Y^{1,T^0}(k) \,\Big|\, Z=1, W, X\Big\} \\
&= E\Big\{Y^{1,0}(k) \,\Big|\, Z=1, W, X\Big\} && \text{One-sided noncompliance} \\
&= \ E\Big\{Y^{1,0}(k) \,\Big|\, H^1(k) \in [k,c], Z=1, W, X\Big\} && \text{Assumption A3-(ii)} \\
&\overset{(\#)}{=} E\Big\{Y^{1,0}(k) \,\Big|\, H^1(k) \in [k,c], T^1(k)=0, Z=1, W, X\Big\} \\
&\overset{(*)}{=} E\Big\{Y^{1,0}(k) \,\Big|\, H^1(k) \in [k,k_m], T^1(k)=0, Z=1, W, X\Big\} \\
&= E\Big\{Y^{1,0}(k) \,\Big|\, H \in [k,k_m], T=0, Z=1, W, X\Big\} && \text{Assumption A2-(i)} \\
&\overset{(*)}{=} E\Big\{Y^{1,0}(k) \,\Big|\, H \in [k,c], T=0, Z=1, W, X\Big\} \\
&= E\Big\{Y(k) \,\Big|\, H \in [k,c], Z=1, W, X\Big\} && \text{Assumption A2-(ii)}
\end{aligned}
$$

  Equality $\overset{(\#)}{=}$ holds from the fact that the event $\{H^1(k) \in [k,c]\} \cap \{Z=1\}$ is the same as the event $\{H^1(k) \in [k,c]\} \cap \{T^1(k)=0\} \cap \{Z=1\}$. Equalities $\overset{(*)}{=}$ hold from the observation that the event $\{H^1(k) \in [k,k_m]\} \cap \{Z=1\}$ is the same as the event $\{H^1(k) \in [k,c]\} \cap \{Z=1\}$ and the event $\{H \in [k,k_m]\} \cap \{Z=1\}$ is the same as the event $\{H \in [k,c]\} \cap \{Z=1\}$.

- (Case 2) For $k \in (c, k_m]$: We have $Y^{1,0}(k) = Y^{1,0}(c)$. Therefore, by following the same algebraic steps as in (Case 1), we obtain:

$$
\begin{aligned}
& E\Big\{Y^{1,T^0}(k) \,\Big|\, Z=1, W, X\Big\} \\
&= E\Big\{Y^{1,T^0}(c) \,\Big|\, Z=1, W, X\Big\} \\
&= E\Big\{Y(c) \,\Big|\, H=c, Z=1, W, X\Big\} \ .
\end{aligned}
$$

Combining (Case 1) and (Case 2), we have the following result for $k \in [k_0, k_m]$:

$$E\Big\{Y^{1,T^0}(k)\,\Big|\,Z=1,W,X\Big\} = E\Big\{Y(k \wedge c)\,\Big|\,H \in [k \wedge c, c], Z=1,W,X\Big\}\ . \tag{S2}$$

Therefore, we have

$$\begin{aligned}
&E\Big\{Y^{1,T^0}(k)\,\Big|\,X\Big\} \\
&= E\Big\{Y^{1,T^0}(k)\,\Big|\,Z=1,X\Big\} && \text{Assumption A3-(i)} \\
&= E\Big[E\Big\{Y^{1,T^0}(k)\,\Big|\,Z=1,W,X\Big\}\,\Big|\,Z=1,X\Big] && \text{Law of iterated expectation} \\
&= E\Big[E\Big\{Y(k \wedge c)\,\Big|\,H \in [k \wedge c, c], Z=1,W,X\Big\}\,\Big|\,Z=1,X\Big]\ . && \text{From (S2)}
\end{aligned} \tag{S3}$$

Lastly, we establish identification of $E\{Y^{1,T^1}(k)\,|\,X\}$. For $k \in [k_0, k_m]$. Let ϵ be the minimum unit of time measurement. Then, we have

$$\begin{aligned}
&E\Big\{Y^{1,1}(k)\,\Big|\,Z=1,W,X\Big\} && \text{Law of iterated expectation} \\
&= E\Big\{Y^{1,1}(k)\,\Big|\,H^1(k) \in [k \vee c + \epsilon, k_m], Z=1,W,X\Big\} && \text{Assumption A3-(ii)} \\
&\overset{(*)}{=} E\Big\{Y^{1,1}(k)\,\Big|\,H^1(k) \in [k \vee c + \epsilon, k_m], T^1(k)=1, Z=1,W,X\Big\} \\
&= E\Big\{Y^{1,1}(k)\,\Big|\,H \in [k \vee c + \epsilon, k_m], T=1, Z=1,W,X\Big\} && \text{Assumption A2-(i)} \\
&= E\Big\{Y(k)\,\Big|\,H \in [k \vee c + \epsilon, k_m], T=1, Z=1,W,X\Big\} && \text{Assumption A2-(ii)} \\
&= E\Big\{Y(k)\,\Big|\,H \in [k \vee c + \epsilon, k_m], Z=1,W,X\Big\}
\end{aligned} \tag{S4}$$

Here, $a \vee b = \max(a,b)$. Equality $\overset{(*)}{=}$ holds from the fact that the event $\{H^1(k) \in [k, k_m]\} \cap \{Z=1\}$ is the same as the event $\{H^1(k) \in [k, k_m]\} \cap \{T^1 = 1\} \cap \{Z=1\}$.

Therefore, for $k \in [k_0, k_m]$:

$$\begin{aligned}
&E\{Y^{1,T^1}(k) \mid X\} \\
&= E[E\{Y^{1,T^1}(k) \mid T^1, W, X\} \mid X] && \text{Law of iterated expectation} \\
&= \sum_w \begin{bmatrix} E\{Y^{1,1}(k) \mid T^1 = 1, W = w, X\} \\ \times P(T^1 = 1, W = w \mid X) \\ +E\{Y^{1,0}(k) \mid T^1 = 0, W = w, X\} \\ \times P(T^1 = 0, W = w \mid X) \end{bmatrix} \\
&= \sum_w \begin{bmatrix} E\{Y^{1,1}(k) \mid Z = 1, T^1 = 1, W = w, X\} \\ \times P(T^1 = 1, W = w \mid Z = 1, X) \\ +E\{Y^{1,0}(k) \mid Z = 1, T^1 = 0, W = w, X\} \\ \times P(T^1 = 0, W = w \mid Z = 1, X) \end{bmatrix} && \text{Assumption A3-(i)} \\
&= \sum_w \begin{bmatrix} E\{Y^{1,1}(k) \mid Z = 1, W = w, X\} \\ \times P(T = 1, W = w \mid Z = 1, X) \\ +E\{Y^{1,0}(k) \mid Z = 1, W = w, X\} \\ \times P(T = 0, W = w \mid Z = 1, X) \end{bmatrix} && \text{Assumptions A2-(i) and A3-(ii)} \\
&= \sum_w \begin{bmatrix} E\{Y(k) \mid H \in [k \vee c + \epsilon, k_m], Z = 1, W = w, X\} \\ \times P(T = 1, W = w \mid Z = 1, X) \\ +E\{Y(k \wedge c) \mid H \in [k \wedge c, c], Z = 1, W = w, X\} \\ \times P(T = 0, W = w \mid Z = 1, X) \end{bmatrix}. && \text{From (S2) and (S4)} \qquad \text{(S5)}
\end{aligned}$$

Based on these identification results, it is straightforward to establish identification of the conditional FNDE and FNIE for $k \in [k_0, k_m]$ as follows:

$$\begin{aligned}
&\tau_{\text{NDE}}(k, X) && \text{(S6)} \\
&= E\Big\{Y^{1,T^0}(k) - Y^{0,T^0}(k) \,\Big|\, X\Big\} \\
&= \text{(S3)} - \text{(S1)} \\
&= E\Big[E\Big\{Y(k \wedge c) \,\Big|\, H \in [k \wedge c, c], Z = 1, W, X\Big\} \,\Big|\, Z = 1, X\Big]
\end{aligned}$$

$$- E\Big[E\Big\{Y(k \wedge c)\,\Big|\, H \in [k \wedge c, c], Z = 0, W, X\Big\}\,\Big|\, Z = 0, X\Big]$$

$$= \sum_w \left[\begin{array}{c} E\Big\{Y(k \wedge c)\,\Big|\, H \in [k \wedge c, c], Z = 1, W = w, X\Big\}P\Big(W = w\,\Big|\, Z = 1, X\Big) \\ -E\Big\{Y(k \wedge c)\,\Big|\, H \in [k \wedge c, c], Z = 0, W = w, X\Big\}P\Big(W = w\,\Big|\, Z = 0, X\Big) \end{array}\right],$$

and

$$\tau_{\text{NIE}}(k, X) \tag{S7}$$
$$= E\Big\{Y^{1,T^1}(k) - Y^{1,T^0}(k)\,\Big|\, X\Big\}$$
$$= \text{(S5)} - \text{(S3)}$$
$$= \sum_w \left[\begin{array}{c} E\Big\{Y(k)\,\Big|\, H \in [k \vee c + \epsilon, k_m], Z = 1, W = w, X\Big\}P\Big(T = 1, W = w\,\Big|\, Z = 1, X\Big) \\ +E\Big\{Y(k \wedge c)\,\Big|\, H \in [k \wedge c, c], Z = 1, W = w, X\Big\}P\Big(T = 0, W = w\,\Big|\, Z = 1, X\Big) \\ -E\Big\{Y(k \wedge c)\,\Big|\, H \in [k \wedge c, c], Z = 1, W = w, X\Big\}P\Big(W = w\,\Big|\, Z = 1, X\Big) \end{array}\right]$$
$$= \sum_w \left[\begin{array}{c} E\Big\{Y(k)\,\Big|\, H \in [k \vee c + \epsilon, k_m], Z = 1, W = w, X\Big\} \\ -E\Big\{Y(k \wedge c)\,\Big|\, H \in [k \wedge c, c], Z = 1, W = w, X\Big\} \end{array}\right] P\Big(T = 1, W = w\,\Big|\, Z = 1, X\Big)\,.$$

Of note, when W is empty, i.e., no post-treatment confounder, the identifying fomulas in (S6) and (S7) reduce to:

$$\tau_{\text{NDE}}(k, X) = E\Big\{Y(k \wedge c)\,\Big|\, H \in [k \wedge c, c], Z = 1, X\Big\} - E\Big\{Y(k \wedge c)\,\Big|\, H \in [k \wedge c, c], Z = 0, X\Big\}$$

and

$$\tau_{\text{NIE}}(k, X) = \left[\begin{array}{c} E\Big\{Y(k)\,\Big|\, H \in [k \vee c + \epsilon, k_m], Z = 1, X\Big\} \\ -E\Big\{Y(k \wedge c)\,\Big|\, H \in [k \wedge c, c], Z = 1, X\Big\} \end{array}\right] P\Big(T = 1\,\Big|\, Z = 1, X\Big)\,.$$

Lastly, (S-)FNDE and (S-)FNIE are identified by marginalizing (S6) and (S7),

respectively. For example, the FNDE and S-FNDE are represented by

$$\tau_{\text{NDE}}(k) = E\Big\{\tau_{\text{NIE}}(k, X)\Big\} \ , \qquad \tau_{\text{NDE}}(k|V=v) = \frac{E\Big\{\mathbb{1}(V=v)\tau_{\text{NDE}}(k, X)\Big\}}{\Pr(V=v)} \ .$$

The FNIE and S-FNIE are similarly represented.

S2 Assumptions When W Is Absent

We introduce a set of modified assumptions necessary to establish identification in the absence of W (i.e., in the presence of X only).

Assumption S1 (Consistency)**.**

(i) If $Z = z$ *and* $k_0 \leq k$*, then* $T = T^z(k)$ *and* $\mathbb{1}(k \leq H) = \mathbb{1}\{k \leq H^z(k)\}$*;*

(ii) If $Z = z$ *and* $T = t$ *and* $k \leq H$*, then* $Y(k) = Y^{z,t}(k)$*.*

Assumption S2 (Unconfoundedness)**.** *For all* $z \in \{0, 1\}$*:*

(i) $Y^{z,t}(\mathcal{K}), H^z(\mathcal{K}) \perp\!\!\!\perp Z \mid X$*;*

(ii) $Y^{z,t}(\mathcal{K}) \perp\!\!\!\perp H^z(\mathcal{K}) \mid (Z = z, X)$*.*

Assumption S3 (Positivity)**.**

(i) $P_{Z|X}(z \mid x) > 0$ *for all* z, x*;*

(ii) $P_{H|Z,X}(h \mid z = 1, x) > 0$ *for all* x *and* $h \in \mathcal{K}$*;*

(iii) $P_{H|Z,X}(h \mid z = 0, x) > 0$ *for all* x *and* $h \in [k_0, c]$*.*

S3 Identifying Assumptions for a General Case

Consider a general case where the mediator T is binary and static, and is not derived from another variable. Also, assume that each subject has measurements available across the entire functional domain $\mathcal{K}$. We can use the counterfactual definitions of the FNDE and FNIE, as well as their subgroup effects, as in Section 4.1.

In this general case, we outline the assumptions required to identify the effects, depending on whether the relationship between Z and T is one-sided or two-sided. For one-sided noncompliance, $T^1 \in \{0, 1\}$ and $T^0 = 0$, while for two-sided noncompliance, $T^z \in \{0, 1\}, z = 0, 1$.

We make the following identifying assumptions for two-sided noncompliance:

Assumption S4 (Consistency)**.**

(i) If $Z = z$, then $T = T^z$;

(ii) If $Z = z$ and $T = t$, then $Y(k) = Y^{z,t}(k)$;

(iii) If $Z = z$, then $W = W^z$.

Assumption S5 (Unconfoundedness)**.** *For any $z \in \{0, 1\}$:*

(i) $Y^{z,t}(\mathcal{K}), T^z, W^z \perp\!\!\!\perp Z \mid X$; (ii) $Y^{z,t}(k) \perp\!\!\!\perp T^z \mid (Z = z, X, W)$

Assumption S6 (Positivity)**.**

(i) $P_{Z|X}(z \mid x) > 0$ for all (z, x);

(ii) $P_{TW|Z,X}(t, w \mid z, x) > 0$ for all (z, t, w, x).

Assumption S7 (Cross-world Independence)**.** *$Y^{z,t}(\mathcal{K}) \perp\!\!\!\perp T^{z'} \Big| (X, W)$ for all $(z, z', t) \in \{0, 1\}^{\otimes 3}$.*

Under one-sided noncompliance (i.e., $T^0 = 0$), Assumption S6-(ii) can be relaxed to only require that (ii-1) $P_{TW|Z,X}(t, w \mid z = 1, x) > 0$ for all (t, w, x) and (ii-2)

$P_{TW|Z,X}(t=0,w \mid z=0,x) > 0$ for all (w,x); moreover, Assumption S7 is no longer needed.

Under Assumptions S4-S7, we establish identification for the FNDE and FNIE. In order to do so, we first establish identification of $E\{Y^{z,t}(k) \mid Z=z,W,X\}$ as follows:

$$
\begin{aligned}
& E\Big\{Y^{z,t}(k) \,\Big|\, Z=z,W,X\Big\} \\
& = E\Big\{Y^{z,t}(k) \,\Big|\, Z=z,T^z=t,W,X\Big\} && \text{Assumption S5-(ii)} \\
& = E\Big\{Y^{z,t}(k) \,\Big|\, Z=z,T=t,W,X\Big\} && \text{Assumption S4-(i)} \\
& = E\Big\{Y(k) \,\Big|\, Z=z,T=t,W,X\Big\} \ . && \text{Assumption S4-(ii)} \qquad \text{(S8)}
\end{aligned}
$$

Consequently, we have the following identification result for a fixed $(z,t) \in \{0,1\}^{\otimes 2}$:

$$
\begin{aligned}
& E\Big\{Y^{z,t}(k) \,\Big|\, X\Big\} \\
& = E\Big\{Y^{z,t}(k) \,\Big|\, Z=z,X\Big\} && \text{Assumption S5-(i)} \\
& = E\Big[E\Big\{Y^{z,t}(k) \,\Big|\, Z=z,W,X\Big\} \,\Big|\, Z=z,X\Big] && \text{Law of iterated expectation} \\
& = E\Big[E\Big\{Y(k) \,\Big|\, T=t,Z=z,W,X\Big\} \,\Big|\, Z=z,X\Big] \ . && \text{Result (S8)} \qquad \text{(S9)}
\end{aligned}
$$

Next, we establish identification of $E\{Y^{z,T^z}(k) \mid X\}$ as follows:

$$
\begin{aligned}
& E\Big\{Y^{z,T^z}(k) \,\Big|\, X\Big\} \\
& = E\Big[E\Big\{Y^{z,T^z}(k) \,\Big|\, T^z,W,X\Big\} \,\Big|\, X\Big] \\
& = \sum_{t,w} E\Big\{Y^{z,t}(k) \,\Big|\, T^z=t,W=w,X\Big\} P\Big(T^z=t,W=w \,\Big|\, X\Big) \\
& = \sum_{t,w} \left[\begin{array}{c} E\Big\{Y^{z,t}(k) \,\Big|\, Z=z,T^z=t,W=w,X\Big\} \\ \times P\Big(T^z=t,W=w \,\Big|\, Z=z,X\Big) \end{array} \right] && \text{Assumption S5-(i)} \\
& = \sum_{t,w} \left[\begin{array}{c} E\Big\{Y^{z,t}(k) \,\Big|\, Z=z,T^z=t,W=w,X\Big\} \\ \times P\Big(T=t,W=w \,\Big|\, Z=z,X\Big) \end{array} \right] && \text{Assumption S4-(i)}
\end{aligned}
$$

$$= \sum_{t,w} \left[\begin{array}{l} E\Big\{Y^{z,t}(k) \,\Big|\, Z = z, W = w, X\Big\} \\ \qquad \times P\Big(T = t, W = w \,\Big|\, Z = z, X\Big) \end{array} \right] \qquad \text{Assumption S5-(ii)}$$

$$= \sum_{t,w} \left[\begin{array}{l} E\Big\{Y(k) \,\Big|\, Z = z, T = t, W = w, X\Big\} \\ \qquad \times P\Big(T = t, W = w \,\Big|\, Z = z, X\Big) \end{array} \right]. \qquad \text{Result (S8)}$$

Next, we establish identification of $E\{Y^{1,T^0}(k) \mid X\}$ as follows:

$$\begin{aligned}
& E\Big\{Y^{1,T^0}(k) \,\Big|\, X\Big\} \\
&= E\Big[E\Big\{Y^{1,T^0}(k) \,\Big|\, T^0, W, X\Big\} \,\Big|\, X\Big] \\
&= \sum_{t,w} E\Big\{Y^{1,t}(k) \,\Big|\, T^0 = t, W = w, X\Big\} P\Big(T^0 = t, W = w \,\Big|\, X\Big) \\
&= \sum_{t,w} E\Big\{Y^{1,t}(k) \,\Big|\, T^1 = t, W = w, X\Big\} P\Big(T^0 = t, W = w \,\Big|\, X\Big) && \text{Assumption S7} \\
&= \sum_{t,w} \left[\begin{array}{l} E\Big\{Y^{1,t}(k) \,\Big|\, Z = 1, T^1 = t, W = w, X\Big\} \\ \qquad \times P\Big(T^0 = t, W = w \,\Big|\, Z = 0, X\Big) \end{array} \right] && \text{Assumption S5-(i)} \\
&= \sum_{t,w} \left[\begin{array}{l} E\Big\{Y^{1,t}(k) \,\Big|\, Z = 1, T^1 = t, W = w, X\Big\} \\ \qquad \times P\Big(T = t, W = w \,\Big|\, Z = 0, X\Big) \end{array} \right] && \text{Assumption S4-(i)} \\
&= \sum_{t,w} \left[\begin{array}{l} E\Big\{Y^{1,t}(k) \,\Big|\, Z = 1, W = w, X\Big\} \\ \qquad \times P\Big(T = t, W = w \,\Big|\, Z = 0, X\Big) \end{array} \right] && \text{Assumption S5-(ii)} \\
&= \sum_{t,w} \left[\begin{array}{l} E\Big\{Y(k) \,\Big|\, Z = 1, T = t, W = w, X\Big\} \\ \qquad \times P\Big(T = t, W = w \,\Big|\, Z = 0, X\Big) \end{array} \right]. && \text{Result (S8)}
\end{aligned}$$

Note that under one-sided noncompliance (i.e., $T^0 = 0$), the cross-world independence assumption (Assumption S7) is not required, as we have established an identification formula for $E\{Y^{1,T^0}(k) \mid X\} = E\{Y^{1,0}(k) \mid X\}$ has already been established in (S9).

Based on these identification results, it is straightforward to establish identification of

the conditional FNDE and FNIE as follows:

$$\begin{aligned}
&\tau_{\text{NDE}}(k, X) \\
&= E\Big\{Y^{1,T^0}(k) - Y^{0,T^0}(k) \,\Big|\, X\Big\} \\
&= \sum_w \left[\begin{array}{l}
E\Big\{Y(k) \,\Big|\, T=1, Z=1, W=w, X\Big\} P\Big(T=1, W=w \,\Big|\, Z=0, X\Big) \\
+E\Big\{Y(k) \,\Big|\, T=0, Z=1, W=w, X\Big\} P\Big(T=0, W=w \,\Big|\, Z=0, X\Big) \\
-E\Big\{Y(k) \,\Big|\, T=1, Z=0, W=w, X\Big\} P\Big(T=1, W=w \,\Big|\, Z=0, X\Big) \\
-E\Big\{Y(k) \,\Big|\, T=0, Z=0, W=w, X\Big\} P\Big(T=0, W=w \,\Big|\, Z=0, X\Big)
\end{array}\right]
\end{aligned}$$

and

$$\begin{aligned}
&\tau_{\text{NIE}}(k, X) \\
&= E\Big\{Y^{1,T^1}(k) - Y^{1,T^0}(k) \,\Big|\, X\Big\} \\
&= \sum_w \left[\begin{array}{l}
E\Big\{Y(k) \,\Big|\, T=1, Z=1, W=w, X\Big\} P\Big(T=1, W=w \,\Big|\, Z=1, X\Big) \\
+E\Big\{Y(k) \,\Big|\, T=0, Z=1, W=w, X\Big\} P\Big(T=0, W=w \,\Big|\, Z=1, X\Big) \\
-E\Big\{Y(k) \,\Big|\, T=1, Z=1, W=w, X\Big\} P\Big(T=1, W=w \,\Big|\, Z=0, X\Big) \\
-E\Big\{Y(k) \,\Big|\, T=0, Z=1, W=w, X\Big\} P\Big(T=0, W=w \,\Big|\, Z=0, X\Big)
\end{array}\right].
\end{aligned}$$

Under one-sided noncompliance (i.e., $T^0 = 0$), these identifying formulas reduce to:

$$\tau_{\text{NDE}}(k, X) = \sum_w \left[\begin{array}{l}
E\Big\{Y(k) \,\Big|\, T=0, Z=1, W=w, X\Big\} \\
-E\Big\{Y(k) \,\Big|\, T=0, Z=0, W=w, X\Big\}
\end{array}\right] P\Big(W=w \,\Big|\, Z=0, X\Big)$$

and

$$\begin{aligned}
&\tau_{\text{NIE}}(k, X) \\
&= \sum_w \left[\begin{array}{l}
E\Big\{Y(k) \,\Big|\, T=1, Z=1, W=w, X\Big\} P\Big(T=1, W=w \,\Big|\, Z=1, X\Big) \\
+E\Big\{Y(k) \,\Big|\, T=0, Z=1, W=w, X\Big\} P\Big(T=0, W=w \,\Big|\, Z=1, X\Big) \\
-E\Big\{Y(k) \,\Big|\, T=0, Z=1, W=w, X\Big\} P\Big(W=w \,\Big|\, Z=0, X\Big)
\end{array}\right].
\end{aligned}$$

Therefore, (S-)FNDE and (S-)FNIE are identified by marginalizing (S6) and (S7), respectively. For example, the FNDE and S-FNDE are represented by

$$\tau_{\mathrm{NDE}}(k) = E\Big\{\tau_{\mathrm{NIE}}(k, X)\Big\} \ , \qquad \tau_{\mathrm{NDE}}(k|V = v) = \frac{E\Big\{\mathbb{1}(V = v)\tau_{\mathrm{NDE}}(k, X)\Big\}}{\Pr(V = v)} \ .$$

The FNIE and S-FNIE are similarly represented.

We also note that the cross-world assumption S7 is not required for identifying the FTE, $E\{Y^{1,T^1}(k) - Y^{0,T^0}(k)\}$, and the S-FTE, $E\{Y^{1,T^1}(k) - Y^{0,T^0}(k) \,|\, V\}$. This is because the cross-world assumption is only necessary for identifying the cross-world counterfactual mean $E\{Y^{1,T^0}(k) \,|\, X\}$ in settings where one-sided noncompliance does not hold.

S4 Details of the Post-treatment Variable Model Estimation

We provide details on the estimation of the post-treatment variable model $g_{zt}(w, x) = P(T = t, W = w|Z = z, X = x)$ in (6). One approach is to factorize g_{zt} as follows:

$$\begin{aligned} g_{zt}(w, x) &= P(T = t, W = w|Z = z, X = x) \\ &= P(T = t \,\Big|\, Z = z, X = x)P(W = w|T = t, Z = z, X = x) \ . \end{aligned}$$

The first function can be estimated using a GAM after applying to a suitable link function. For instance, a logistic GAM may be specified as:

$$\text{logit}\{P(T = 1 \,|\, Z = z, X = x)\} = \alpha_{0z} + \sum_{j=1}^{p} f_{jz}(x_j) \ , \tag{S10}$$

where f_{jz} is a univariate smooth function of X_j. In practice, these smooth functions are defined similarly to (10), so the estimation of model (S10) parallels the estimation of m_{zt} described in Section 4.3.

The estimation of the second function depends on the type of W. If W is discrete, a GAM with an appropriately chosen link function can be used, in a manner similar to the approach in (S10). However, if W is continuous or multivariate, the estimation becomes more challenging. In such cases, one can specify a likelihood for the conditional distribution $W|(T = t, Z = z, X = x)$ and estimate the corresponding density using maximum likelihood methods. For example, when W is a univariate, continuous variable, one can assume that W follows a normal distribution as follows:

$$W|(T = t, Z = z, X = x) \sim N\Big(\mu_{tz}(x), \sigma^2_{tz}(x)\Big) \ .$$

In this model, the density is fully characterized by the mean function $\mu_{tz}(x)$ and the variance function $\sigma^2_{tz}(x)$, so estimating these two functions suffices. Both $\mu_{tz}(x)$ and $\sigma^2_{tz}(x)$

can be modeled using either simple parametric forms or GAMs, with their parameters estimated by maximizing the likelihood.

S5 Details of the Bootstrap Confidence Interval Construction

We detail the procedure for constructing bootstrap confidence intervals. Let $\mathcal{D}$ be the observed data of n subjects, and B be the number of bootstrap replications. For each bootstrap replication, indexed by $b = 1, \ldots, B$, we draw a bootstrap sample $\mathcal{D}^{[b]}$ by randomly sampling n subjects with replacement from $\mathcal{D}$. Using $\mathcal{D}^{[b]}$, we obtain the FNDE, FNIE, and FTE estimators, denoted by $\hat{\tau}^{[b]}_{\text{NDE}}(k)$, $\hat{\tau}^{[b]}_{\text{NIE}}(k)$, and $\hat{\tau}^{[b]}_{\text{TE}}(k)$, respectively. The 95% bootstrap confidence interval for each causal effect at k is obtained by taking the central 95% quantiles of the corresponding bootstrap estiamtes. The following algorithm summarizes this procedure.

Algorithm 1 Bootstrap Confidence Intervals

Require: Data $\mathcal{D}$, number of bootstrap replications B

```
for b = 1, ..., B do
    D^[b] ← bootstrap sample by randomly drawing n subjects with replacement from D
    τ̂^[b]_NDE(k) ← a FNDE estimator in (12) based on D^[b]
    τ̂^[b]_NIE(k) ← a FNIE estimator in (13) based on D^[b]
    τ̂^[b]_TE(k) ← a FTE estimator in (14) based on D^[b]
end for
for k = k_0, k_0 + 1, ... do
    CI_NDE(k) ← the central 95% quantile of {τ̂^[1]_NDE(k), ..., τ̂^[B]_NDE(k)}
    CI_NIE(k) ← the central 95% quantile of {τ̂^[1]_NIE(k), ..., τ̂^[B]_NIE(k)}
    CI_TE(k) ← the central 95% quantile of {τ̂^[1]_TE(k), ..., τ̂^[B]_TE(k)}
end for
return The 95% bootstrap confidence intervals {CI_NDE(k) | k_0 ≤ k}, {CI_NIE(k) | k_0 ≤ k},
    {CI_TE(k) | k_0 ≤ k}
```

S6 Details of the Implementation of the Simulation Study

We used `R` (R Core Team, 2024) to implement both the simulation study and data analysis. The implementation details for the simulation study are provided as an example, while the data analysis followed a similar approach.

Let `Data` be an `R` data frame generated during the simulation study, with the following structure, where Z, T, W, X_1, and X_2 are all binary variables.

ID	Y	K	Z	T	W	X1	X2
1	2	6	0	0	1	1	0
⋮	⋮	⋮	⋮	⋮	⋮	⋮	⋮
1	70	15	0	0	1	1	0
2	1	6	1	0	0	0	0
⋮	⋮	⋮	⋮	⋮	⋮	⋮	⋮
2	88	15	1	0	0	0	0
N	3	6	1	1	1	0	1
⋮	⋮	⋮	⋮	⋮	⋮	⋮	⋮
N	93	40	1	1	1	0	1

Then, $m_{zt}(k, w, x) = E\{Y^{z,t}(k)|Z = z, W = w, X = x\}$ is estimated by the following `R` command using the `mgcv` package.

```
gam(formula = Y ~ X1 + X2 + W +
                  s(K,k=knots) + s(K,by=X1,k=knots) +
                  s(K,by=X2,k=knots) + s(K,by=W,k=knots),
    family = gaussian(),
    gamma = gamma,
    data = Data[Data$Z==z & Data$T==t,])
```

Here, `knots` is the number of knots used for the thin plate basis function, and `gamma` is the amount of smoothing to apply to the spline curves. These choices can be further checked via `gam.check` command. In both the simulation study and data analysis, we selected

`knots`=10 and `gamma`=1, which resulted in no diagnostic issue.

For the linear and cubic estimator, $m_{zt}(k, w, x)$ was estimated based on the following specification:

Linear Estimator :
```
lm(formula = Y ~ X1 + X2 + W +
                      K + K:X1 + K:X2 + K:W,
     data = Data[Data$Z==z & Data$T==t,])
```
Cubic Estimator :
```
lm(formula = Y ~ X1 + X2 + W +
                      K + I(K^2) + I(K^3) + K:X1 + K:X2 + K:W,
     data = Data[Data$Z==z & Data$T==t,]) .
```

Specifically, the basis functions in (9) were specified as $\phi_{zt,l}(k) = (1, k)$ for the linear estimator and $\phi_{zt,l}(k) = (1, k, k^2, k^3)$ for the cubic estimator. Also, the basis functions in (10) and (11) were specified as $\phi_{zt,l}(s_j) = s_j$, and $\phi_{zt,l}(k, s_j) = ks_j$, respectively.

In order to estimate the post-treatment variable model $g_{zt}(w, x)$, we used the following decomposition:

$$\begin{aligned} g_{zt}(w, x) &= P(T = t, W = w|Z = z, X = x) \\ &= P(T = t \Big| Z = z, X = x)P(W = w|T = t, Z = z, X = x) \ . \end{aligned}$$

For the propensity score model $\Pr(T \mid Z, X)$, it suffices to estimate $\Pr(T = t \mid Z = 1, X)$ due to one-sided noncompliance, i.e., $\Pr(T = 0 \mid Z = 0, X) = 1$. Specifcally,
$\Pr(T = 1 \mid Z = 1, X)$ is estimated by

```
gam(formula = T ~ X1*X2,
    family = binomial(),
    gamma = gamma,
```

```
data = Data[Data$Z==1,]) .
```

Next, the post-treatment confounder model, $P(W \mid T, Z, X)$, can similarly be estimated because W is binary. Specifically, we used the following model for $(z,t) \in \{(0,0),(1,0),(1,1)\}$:

```
gam(formula = W ~ X1*X2,
    family = binomial(),
    gamma = gamma,
    data = Data[Data$Z==z & Data$T==t,]) .
```

S7 Description of Variables

Table S1

Description of Variables

Type	Variables	Description
Outcome Y	Functional test scores	Cumulative test scores over time
	Functional item access	Cumulative number of accessed test items over time
Treatment Z	ETA receipt	Whether the student received ETA (No = 0; Yes = 1)
Mediator T	ETA use	Whether the student used ETA (No = 0; Yes = 1) $T = \mathbb{1}(H > c)$, where H = observed total response time and c = standard testing time
Pre-treatment Covariate X	Sex	Student's sex (Male = 0; Female =1)
	Race	Student's race/ethnicity (White = 0; African American = 1; Hispanic = 2; Others = 3)
	PARED	Parents' education level (High school =0; Grad college = 1; I do not know = 2; Omitted and missing = 3)
	IEP	Whether this student has disabilities or not (No = 0; Yes = 1)
	SLunch	Whether this student is eligible for National School Lunch Program (Others = 0; Eligible = 1)
	ClassNextYear	Student's math course planned to take next year (Basic Math = 0; Algebra I = 1; Geometry = 2; Algebra II = 3; I do not know = 4; Omitted and missing = 5)
	Interest	Student's interest in math (Low = 0; Moderate = 1; High = 2, Missing = 3)
Post-treatment Covariate W	Early Item Access	Student's number of items accessed by 6 minutes exceeds the sample median (Low = 0; High = 1)

NOTE: ETA = Extended time accommodation.

SOURCE: U.S. Department of Education, National Center on Educational Statistics (NCES), 2017 NAEP Grade 8 Mathematics Process Data, Student Features Data File Partial Form and Response Data File.
